# Fast Programming of in‐Plane Hyperbolic Phonon Polariton Optics through Van der Waals Crystals Using the Phase‐Change Material In_3_SbTe_2_

**DOI:** 10.1002/advs.77633

**Published:** 2026-09-08

**Authors:** Lina Jäckering, Umberto Saldarelli, Aaron Moos, Lukas Conrads, Enrique Terán‐García, Christian Lanza, Aitana Tarazaga Martín‐Luengo, Gonzalo Álvarez‐Pérez, Pablo Alonso‐González, Matthias Wuttig, Thomas Taubner

**Affiliations:** ^1^ First Institute of Physics (IA) RWTH Aachen University Aachen Germany; ^2^ Juelich‐Aachen Research Alliance (JARA‐FIT) Juelich Germany; ^3^ Department of Physics University of Oviedo Oviedo Spain; ^4^ Center of Research on Nanomaterials and Nanotechnology CINN (CSIC‐Universidad De Oviedo) El Entrego Spain; ^5^ Istituto Italiano di Tecnologia Center for Biomolecular Nanotechnologies Arnesano Italy

**Keywords:** fabrication, laser, materials science, nanostructure, near field optical microscope, phase‐change material, phonon, polariton, hyperbolic polariton, van der waals heterostructures

## Abstract

The high directionality of hyperbolic phonon polaritons (HPhPs) has opened radically new ways to route and steer the flow of energy at the nanoscale. However, launching HPhPs requires fabricating efficient and precisely aligned polariton launching structures, what remains time‐consuming and expensive with conventional nanofabrication approaches. Recently, using visible laser pulses, polariton launching structures have been programmed into the plasmonic phase‐change material In_3_SbTe_2_. Here, we leverage this approach to tailor in‐plane hyperbolic polaritons by programming launching and confining nanostructures through α‐MoO_3_ flakes deposited onto In_3_SbTe_2_. Importantly, optical programming after flake deposition enables alignment of launching stripes to the flake's [001]‐axis, essential to control the directional polariton propagation. We showcase i) an optically programmed focusing disk, showing similar tuning ranges and confinement as focusing by gold disks; and ii) a first experimental realization toward a nanocavity for in‐plane HPhPs created by reconfiguring the single disk to a double disk structure, tailoring the confinement by simply reprogramming the disk distance. Our fabrication scheme offers fast turnaround times, flexible alignment, and the opportunity to reversibly reconfigure structures within 30 s. Thus, it is a fast, efficient, and versatile way to tailor propagation and confinement of highly directional polaritons on demand.

## Graphical abstract

Launching structures for in‐plane hyperbolic polaritons in α‐MoO_3_ are optically programmed through already deposited flakes into the phase‐change material In_3_SbTe_2_. Precise alignment with the flake's crystal axes, focusing of polaritons by an optically programmed disk, and a first experimental realization toward a nanocavity for in‐plane hyperbolic polaritons created by reconfiguring the single disk to a double disk structure are demonstrated.

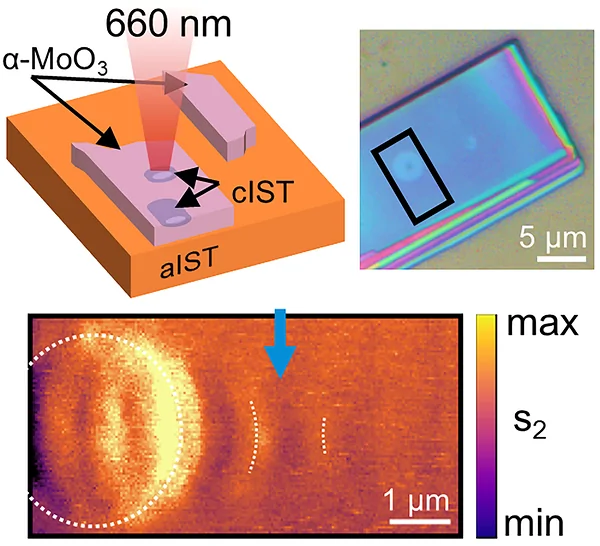

## 1 Introduction

Polaritons—hybridized excitations of photons and collective, dipolar oscillations in a material—in van der Waals (vdW) materials are prone to high confinement and therefore prime candidates for nanophotonic devices. VdW materials host a plethora of polaritons of different kinds [1]; among those are surface plasmon polaritons in graphene [2, 3], out‐of‐plane hyperbolic phonon polaritons in hBN [4], and in‐plane hyperbolic phonon polaritons in α‐MoO_3_ [5, 6, 7, 8]_,_ in α‐V_2_O_5_ [9], or in LiV_2_O_5_ [10]_._ The in‐plane hyperbolic phonon polaritons in α‐MoO_3_ have been demonstrated to show high directionality [11, 12, 13], planar and negative refraction [14, 15, 16, 17], hyperfocusing [15, 18, 19, 20, 21, 22] and negative reflection [23]. These unusual, fundamental optical phenomena at the nanoscale [24] result from the high directionality of the polaritons in α‐MoO_3_. This exceptional high directionality facilitates routing and steering of the polaritons and thereby of the flow of energy at the nanoscale, which makes the highly directional polaritons promising for nanophotonic devices [25].

Steering polaritons and studying fundamental optical phenomena in anisotropic vdW materials requires fabrication of optical structures with precise angular alignment relative to the crystal axes. Conventionally, these optical structures are realized by adding metallic launching structures or by etching away parts of the polariton hosting material [5, 11, 12, 13, 14, 15, 18, 19, 20, 21, 22]. However, these conventional tailoring approaches rely on cumbersome and time‐consuming fabrication techniques, including lithography and etching (c.f. Note S1), which usually take several days. Especially, the fabrication of launching structures to explore in‐plane anisotropic materials such as α‐MoO_3_ requires precise alignment of the vdW material flake with the fabricated structure, as their alignment is crucial for controlling the in‐plane anisotropic propagation. Further, structures realized through conventional fabrication techniques are usually static, offering no possibility for post‐fabrication adaptation.

Tunable polariton optics can be realized by combining α‐MoO_3_ with tunable or programmable substrates since the polariton propagation can be tailored by substrate engineering (c.f. Note S2 for comparative literature study). Global tuning of polariton wavelength and direction have been realized by placing α‐MoO_3_ flakes on different substrates [21] or tailoring the optical properties of the substrate [13, 17, 20]. However, optical structures such as focusing structures for these tunable polaritons still rely on conventional time‐consuming fabrication techniques. Fast prototyping of polariton optics instead has been realized by locally changing the optical conductivity of the oxide SmNiO_3_ and afterward aligning an α‐MoO_3_ flake [26]. While polariton optics have been directly imprinted, this approach has not been used for prototyping after flake deposition nor for reconfiguring polariton optics.

Fast prototyping of reversibly reconfigurable polariton optics can be realized with phase‐change materials (PCMs). PCMs can be reversibly switched between two (meta‐)stable phases, an amorphous and a crystalline phase, by inducing heat, e.g., by visible laser pulses [27, 28]. The two phases do usually feature distinct electronic and optical properties. For example, the two dielectric phases of GeSbTe (GST) alloys typically feature a permittivity contrast of about 24 in the infrared range between 800 and 970 cm^−1^ [27]. The pronounced contrast of the optical properties in PCMs like Ge_2_Sb_2_Te_5_ has been attributed to a change in bonding between the amorphous and crystalline states [28, 29]. While amorphous PCMs employ ordinary covalent bonding, crystalline Ge_2_Sb_2_Te_5_ utilizes a special bonding mechanism called metavalent bonding [30]. This bonding mechanism is characterized by unconventional properties [31] and an unusual bond rupture in atom probe tomography [32]. This permittivity contrast has been exploited to refract hyperbolic phonon polaritons in hBN launched at a flake edge by an optically programmed lens in the GST below [33]. Recently, the PCM In_3_SbTe_2_ (IST) has been introduced for fast prototyping of reconfigurable nanophotonics [34], such as antennas [35, 36, 37, 38, 39] and advanced metasurfaces [40, 41] for emissivity control, beam‐steering, lensing, and holography. The PCM IST stands out as it can be reversibly switched between a dielectric amorphous (ε_aIST_ = 14 > 0) and a metallic crystalline phase (ε_cIST_ = ‐159 < 0) featuring a permittivity contrast of about 173 in the infrared range between 800 and 970 cm^−1^ [35]. Thus, the PCM IST is a promising platform for fast optical prototyping of reconfigurable launching structures for polaritons in the infrared [42, 43, 44]. IST has been used to launch and confine surface phonon polaritons in SiC [42], surface plasmon polaritons in the doped semiconductor CdO [43], and hyperbolic phonon polaritons in hBN [44, 45]. Since most vdW materials are highly transparent in the visible spectral range that is used for optical programming of structures in IST, the structures can be programmed through the already deposited flake [44]. While the modification of the α‐MoO_3_ polariton dispersion by IST as a substrate and optical programming of a focusing structure have already been demonstrated [45], the major advantage of programming polariton optics after flake deposition and their reconfiguration has not been demonstrated yet. Importantly, optical programming after flake deposition allows for alignment of the structures to the flake axes, for reversible reconfiguration of previously written structures, and for addition of structures to the very same flake. Here, we deposit the in‐plane hyperbolic vdW material α‐MoO_3_ onto the phase‐change material IST. After flake deposition, we rapidly prototype polariton optics through the α‐MoO_3_ flake into the IST within 30 s (see Notes S1 and S3). First, we retrieve the experimental dispersion of polaritons launched at a phase boundary of IST below the α‐MoO_3_ flake by performing sequential near‐field optical spectroscopy. We optically program stripes of arbitrary angle with respect to the crystallographic axis of α‐MoO_3_ to steer the high directionality of polaritons in α‐MoO_3_. By optically programming a circular crystalline disk into the IST, we achieve focusing of α‐MoO_3_ 's polaritons that can be tailored by varying the illumination frequency. Finally, we showcase a first experimental realization toward a nanocavity for the in‐plane hyperbolic polaritons by adding a second disk in a post‐processing step. To further tailor the polariton confinement, we vary the disk distance. Our experimental findings, supported by numerical simulations, demonstrate the promising potential that IST holds for on‐demand (re‐)programming of polariton optics for vdW materials within 30 s (see Note S3) and for impacting the next generation of compact nanophotonic devices.

## 2 Results

**FIGURE 1:**
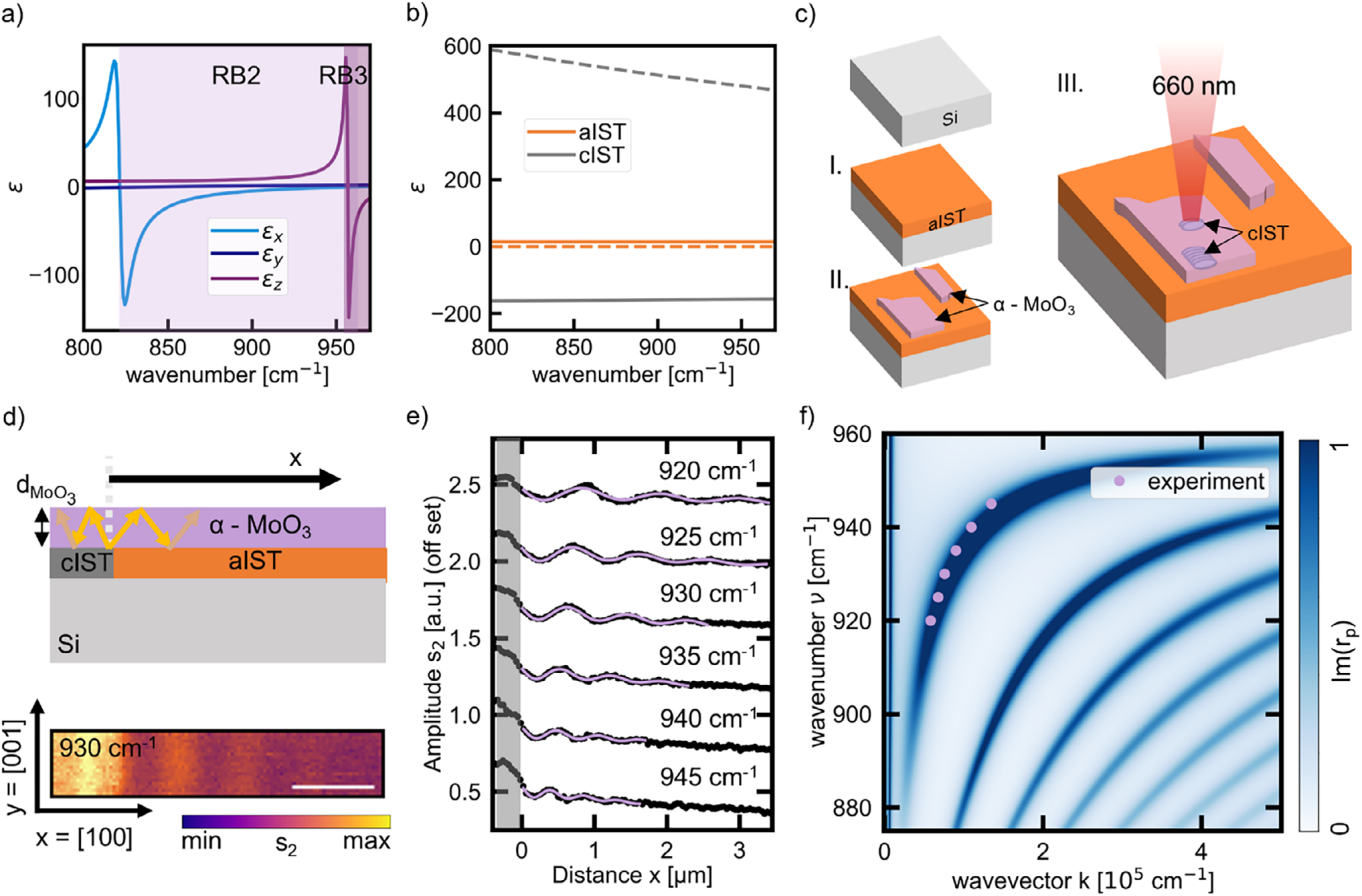
Direct programming of α—MoO_3_ phonon polariton launching structures into In_3_SbTe_2_ (IST). (a) Real part of the permittivity ε of α‐MoO_3_ and (b) real part (solid) and imaginary part (dashed) of the permittivity ε of IST in the reststrahlen band (RB2: 821.4 – 963 cm^−1^) of α‐MoO_3,_ where permittivity along the optical *x*‐axis ε_x_ is negative. Note that RB2 overlaps slightly with RB3 (956.7 – 1006.9 cm^−1^), where the permittivity along the optical *z*‐axis ε_z_ is negative. (c) Schematic layer stack fabrication and subsequent programming of optical structures. (d) Cross‐sectional sketch of the investigated structure programmed into the IST by local optical crystallization to launch phonon polaritons in the α‐MoO_3_ ($d_{\alpha -\text{Mo}O_{3}}$ = 392 nm) above (top). Exemplary s‐SNOM amplitude image of the phonon polaritons launched at the boundary of amorphous and crystalline IST (bottom), extracted from the same images that will be used in Figure 3. The scale bar is 1 µm. (e) Line profiles extracted from s‐SNOM amplitude images at the boundary of amorphous and crystalline IST as in (d) for different illumination frequencies, fitted assuming only edge‐launched polaritons (purple lines). (f) Experimental (purple dots, extracted from the line profiles in (e)) and simulated dispersion (color plot of the imaginary part of the Fresnel reflection coefficient) of the phonon polaritons of α‐MoO_3_ on top of amorphous IST.

Aiming to steer the anisotropic polaritons in α‐MoO_3_, we combine the vdW material flakes with the PCM IST that allows for optical programming of reconfigurable polariton launching optics. α‐MoO_3_ is a highly anisotropic vdW material that has distinct permittivities along all three crystal axes [46]. In the following, we choose the coordinate system such that the *x*‐axis is aligned with the [100], the *y*‐axis with the [001], and the *z*‐axis with the [010] direction of the α‐MoO_3_ crystal. Remarkably, the phonon resonances along the optical *x‐*, *y‐*, and *z*‐axis of the flake occur at different frequencies such that the reststrahlen bands (RBs) – the spectral range between the transverse and the longitudinal optical phonon resonances, where the permittivity is negative – do barely overlap. We will focus on the RB that spans from 821.4 to 963 cm^−1^ (commonly referred to as RB2), where the permittivity along the *x*‐axis is negative, while in most of the spectral range the permittivity along *y*‐ and *z*‐axes is positive [46], as depicted in Figure 1a. Consequently, the isofrequency curve (IFC) in the corresponding Fourier space (k_x_,k_y_) is hyperbolic [47] (see Note S4). Note that also the IFC in (k_x_,k_z_) is hyperbolic. Hence, the polaritons in α‐MoO_3_ are out‐of‐plane hyperbolic and volume‐confined [5, 8, 47, 48], similar to out‐of‐plane hyperbolic phonon polaritons in hBN [4, 49, 50], but additionally they are in‐plane hyperbolic. This leads to strongly anisotropic propagation of polaritons in α‐MoO_3_ [5, 6, 8, 11]. As stated above, IST can be switched between the dielectric amorphous phase (aIST) characterized by a positive permittivity and the metallic crystalline phase (cIST) characterized by a negative permittivity (c.f. Figure 1b). Hence, IST serves as a substrate that can be locally switched between a dielectric and metallic phase and thus offers fast local programming of reconfigurable polariton optics. Thereby, we use a fabrication scheme that overcomes the drawbacks of conventional multistep fabrication processes for polariton optics, which usually take several days (c.f. Note S1). Our fabrication scheme is based only on three steps (c.f. Figure 1c). First, the IST film is sputtered in the amorphous phase onto a silicon wafer, followed by sputtering of a capping layer of ZnS:SiO_2_ to prevent the IST from oxidation (omitted in sketches). Second, α‐MoO_3_ is mechanically exfoliated and transferred (see Methods) onto the IST thin film, resulting in several α‐MoO_3_ flakes of different thicknesses and in‐plane dimensions distributed over the sample. Thirdly, we optically program the desired structure through the α‐MoO_3_ flake into the IST by locally crystallizing the IST using a pulsed laser with a wavelength of 660 nm (see Methods). By spatially overlapping several laser pulses, within 30 s we can program arbitrary shapes of cIST at any position as sketched in Figure 1c. Compared to conventionally used fabrication schemes, we identify three major advantages: 1) The fewer fabrication steps lead to much faster turnaround times. Using our fabrication scheme, the sample fabrication is accomplished within a few hours, and launching structures can be created within 30 s. 2) We can optically program structures after flake deposition. Thus, we can also use the much faster non‐deterministic flake transfer and can align the structure to the flake axes, while in conventional approaches the flake needs to be aligned with the structure. 3) Our fabrication allows for post‐processing adaptation, which we demonstrate in Note S3. We can reconfigure the already written structure and can adapt size and shape by further crystallization or reamorphization. Further, we can add additional structures to the same flake (or other flakes on the same wafer) within 30 s, as we do not need to repeat the first two steps.

We first explore polaritons launched at a phase boundary in the IST below the α‐MoO_3_ as sketched in Figure 1d top. Due to the vast permittivity difference between amorphous and crystalline IST, polaritons are launched at the phase boundary. The hyperbolic polaritons of α‐MoO_3_ are volume‐confined, i.e., the electric field of the polaritonic mode is confined within the thin α‐MoO_3_ slab [51], and propagate through α‐MoO_3_ toward both sides of the boundary. We employ scattering‐type scanning near‐field optical microscopy (s‐SNOM) [52, 53, 54] (see Methods) to image the phase‐boundary‐launched polaritons that interfere with the incident light, similar to previously investigated phase‐boundary‐launched polaritons in hBN [44]. We do not observe tip‐launched and edge‐reflected but only phase‐boundary‐launched polaritons that interfere with the incident light, similar to edge‐launched polaritons [55, 56, 57]. An exemplary s‐SNOM amplitude image at 900 cm^−1^ of a phase boundary below the α‐MoO_3_ is shown in Figure 1d bottom. This image depicts an area of high s‐SNOM amplitude on the left and a lower s‐SNOM amplitude on the right, corresponding to the metallic cIST with highly negative permittivity on the left and the dielectric aIST with a positive permittivity on the right, respectively. Within the area of aIST, we observe bright fringes parallel to the phase boundary. These bright fringes correspond to polaritons launched at the phase boundary and propagating in the α‐MoO_3_ above aIST. To quantify the polariton dispersion, we extract line profiles perpendicular to the phase boundary in the area of aIST from s‐SNOM amplitude images like that in the bottom of Figure 1d for different frequencies (Figure 1e). From the line profiles, we extract the polariton wavevector k_p_ (see Note S5 for details) that corresponds to the polariton wavelength λ_p_ via $k_{p}=\frac{2\pi }{\lambda_{p}}\;$ to determine the experimental dispersion (purple dots in Figure 1f). With increasing frequency, the polariton wavevector (wavelength) increases (decreases). The experimentally observed dispersion agrees with the theoretical prediction from the imaginary part of the Fresnel reflection coefficient (blue branches in Figure 1f) that we calculated using the transfer matrix method [58].

**FIGURE 2:**
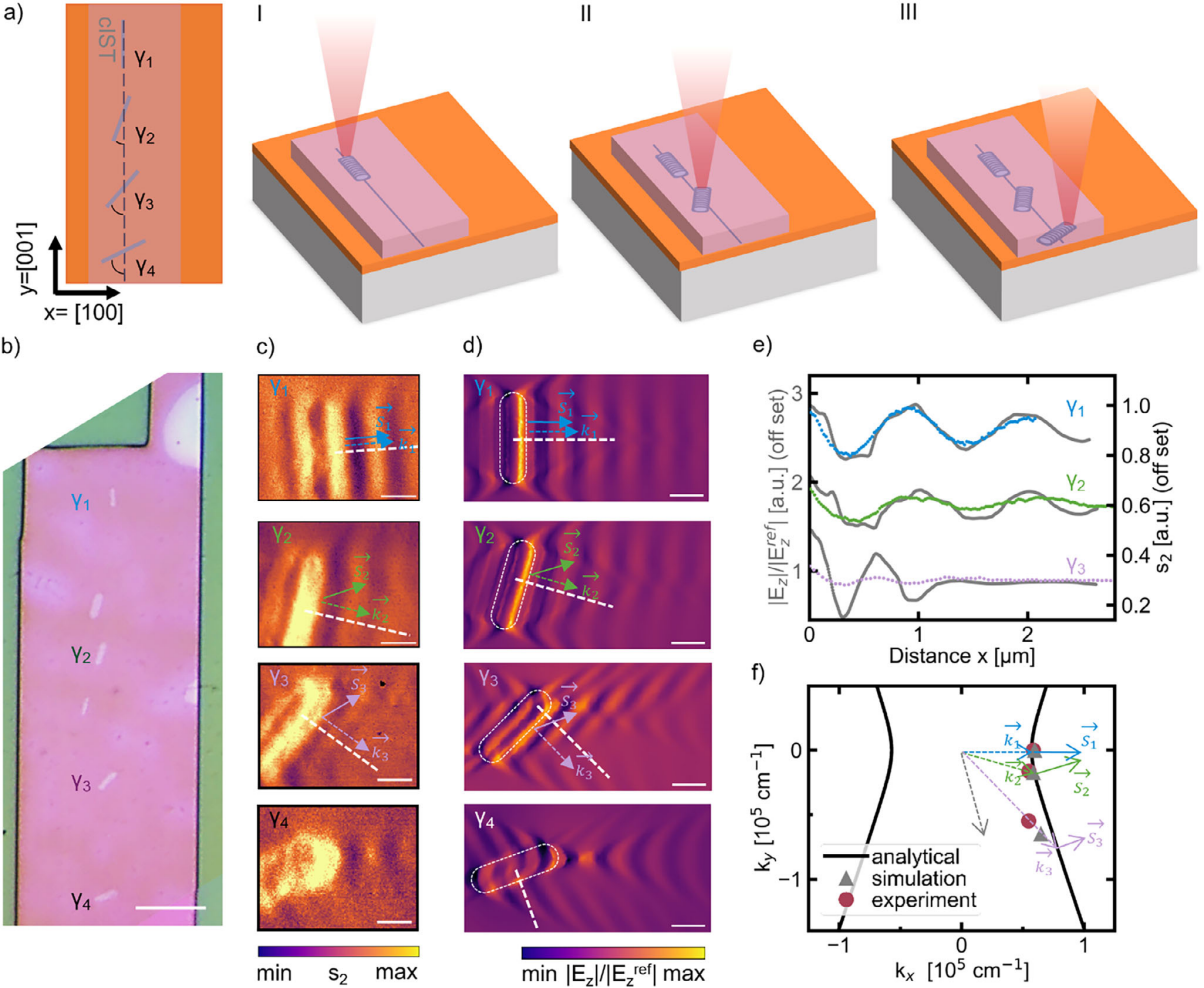
Fast prototyping of launching stripes of different angles with respect to the crystal axis. (a) Design sketch and optical writing process of stripes tilted by different angles with respect to the *y*‐axis of the crystal. (b) Optical microscope image of the tilted antennas programmed into the IST below the α‐MoO_3_ flakes ($d_{\alpha -\text{Mo}O_{3}}$ = 252 nm). The scale bar corresponds to 5 µm. (c) s‐SNOM amplitude images at 900 cm^−1^ and (d) corresponding simulation of antennas with four different tilt angles. The scale bars correspond to 1 µm. In (b)‐(d), the angle between the stripe long axis and the *y*‐axis increases from top to bottom with γ_1_ = ‐2.4°, γ_2_ = 16.5°, γ_3_ = 44.7°, and γ_4_ = 70.2°. The wavefronts are parallel to the long axes of the stripes, and thus the wavevectors are perpendicular to the long axes of the stripes (indicated by the dashed arrows). In contrast, the direction of energy flow (Poynting vector: solid arrows) varies depending on the tilt angle. (e) Line profiles perpendicular to the stripes' long axes extracted from the s‐SNOM amplitude images (color corresponding to the tilt angle as in (b)‐(d)) and from simulations (grey), as indicated by the white dashed lines in (c) and (d), respectively. (f) IFC with wavevectors extracted from experiment (red dots) and simulation (grey triangles). The dashed arrows indicate the wavevector and the solid arrows the Poynting vectors.

Exploiting our fast fabrication scheme that also allows for (re‐)programming of launching structures through already deposited flakes and thus for their flexible alignment with the optical axes of the flake (Figure 2a), we study the in‐plane anisotropic propagation of the polaritons. To investigate the directional propagation, we optically program crystalline launching stripes with different orientations with respect to the *y*‐axis as sketched in Figure 2a. The concept of launching stripes with different orientations is similar to those conducted in the literature using conventionally fabricated metallic launching stripes [13] and etched trenches [11]. The angle γ between the long axis of the stripes and the *y*‐axis is increased sequentially from top to bottom. In the optical light microscope image (Figure 2b), the crystalline stripes appear as bright areas within the pink α‐MoO_3_ flake. We investigate the anisotropic polaritons in α‐MoO_3_ [5, 6, 11] in the RB2, where the permittivity along the *x*‐axis is negative. Thus, from the IFC, which graphically displays the solution of the dispersion relation at a fixed frequency in Fourier space (c.f. Notes S4 and S12), we expect the propagation to be preferential along the *x*‐direction and forbidden along the *y*‐direction, as observed in the literature [5, 6, 11]. The propagation direction is restricted to a cone defined by the complementary angle θ =$\;\frac{\pi }{2}-\alpha$ to the opening angle α of the IFC. Experimentally, we use the s‐SNOM to visualize the propagating polaritons (shown in Figure 2c) and support our experimental results with simulations of the electric field (see Figure 2d).

In Figure 2c top image, the stripe is almost aligned with the *y*‐axis; thus γ_1_∼0°. We observe bright fringes parallel to the long axis of the stripe (*y*‐axis) and periodic in the *x*‐direction. These bright fringes originate from polaritons launched by the cIST stripe and interfering with the incident light. The direction of polariton propagation (perpendicular to the bright fringes; *x*‐direction) corresponds to the direction of the wavevector (indicated by the blue dashed arrow) and is perpendicular to the stripe. Also, the direction of energy flow corresponding to the direction of the Poynting vector (indicated by the blue solid arrow) is perpendicular to the stripe. In this case, the Poynting vector and wavevector are collinear. To further analyze the polariton propagation, we extract line profiles perpendicular to the stripe from both s‐SNOM amplitude and simulation images. The resulting line profiles are shown in Figure 2e. By fitting the line profiles (see Note S5), we experimentally determine the polariton wavelength and, thus, the magnitude of the polariton wavevector. Figure 2f displays a direct comparison between the experimentally determined wavevector (red dots) and the simulation (grey triangles), for each angle γ. The black solid line represents the analytically calculated IFC of the system following ref [59]. (c.f. Note S12). For γ_1_∼0°, the wavevector is parallel to the k_x_‐axis and k_y_ = 0. Considering the IFC, we see for γ_1_∼0° that the Poynting vector, whose direction is always perpendicular to the IFC, is collinear with the wavevector and also along the *x*‐direction, in good agreement with our experimental observations.

For increasing angle γ between the long axis of the stripes and the *y*‐axis in the s‐SNOM amplitude images in Figure 2c and simulations in Figure 2d, we observe bright fringes parallel to the stripe for γ_2_ and γ_3_ again, whereas for γ_4_ no fringes parallel to the long axis of the stripe are observed. The spacing between the fringes decreases with increasing angle γ. While the wavevector of the polaritons (indicated by dashed arrows) launched by the stripes with γ_2_ and γ_3_ is perpendicular to the stripes as it is for γ_1_∼0°, the direction of energy flow (indicated by solid arrows) is no longer perpendicular to the stripe nor collinear with the direction of propagation. For a qualitative understanding, we again extract line profiles (Figure 2e), determine the polariton wavelength, and calculate the magnitude of the polariton wavevector. In agreement with the observation of reduced spacing of the fringes, we find a reduced polariton wavelength for an increased angle γ, corresponding to an increased polariton wavevector. To plot the wavevector in the IFC, the angle of propagation with respect to the *x*‐axis has to be considered, which is given by the angle γ between the long axis of the stripes and the *y*‐axis. For a non‐zero angle γ, the wavevector is no longer aligned with the *x*‐axis; the wavevector component k_y_ is non‐zero. The wavevectors extracted from simulation and experiment agree well and follow the trend of the theoretical IFC. As conducted in the literature [11, 13, 23, 60], by plotting the wavevector in the IFC and considering the propagation direction, the non‐collinearity of the wavevector and Poynting vector can be explained. Since the Poynting vector is normal to the IFC, for any wavevector direction not aligned with the *x*‐axis, the Poynting vector is not collinear with the wavevector. The direction of energy flow and the propagation direction are not collinear, explaining the observations in the s‐SNOM images and simulations.

If the angle γ between the long axis of the stripes and the *y*‐axis exceeds the opening angle of the IFC, the wavevector direction would be such that no intersection with the IFC is possible (see grey dashed arrow in Figure 2f) and thus propagation along this direction is not allowed [11, 13, 23]. We do not observe fringes parallel to the stripe aligned with angle γ_4_ (c.f. Figure 2c) as the angle γ_4_ exceeds the opening angle of the IFC.

Relying on optically programmed launching stripes in IST, we can retrieve the IFC experimentally, thereby characterizing the directionality and, at the same time, steer the direction of polariton propagation similar to previous experiments relying on conventionally fabricated metallic launching stripes [11, 13]. In contrast to conventional fabrication techniques, we can speed up the fabrication process as we drastically decrease the turnaround times and can flexibly align the structures to the flake axes, facilitating characterization and steering of anisotropic polaritons.

**FIGURE 3:**
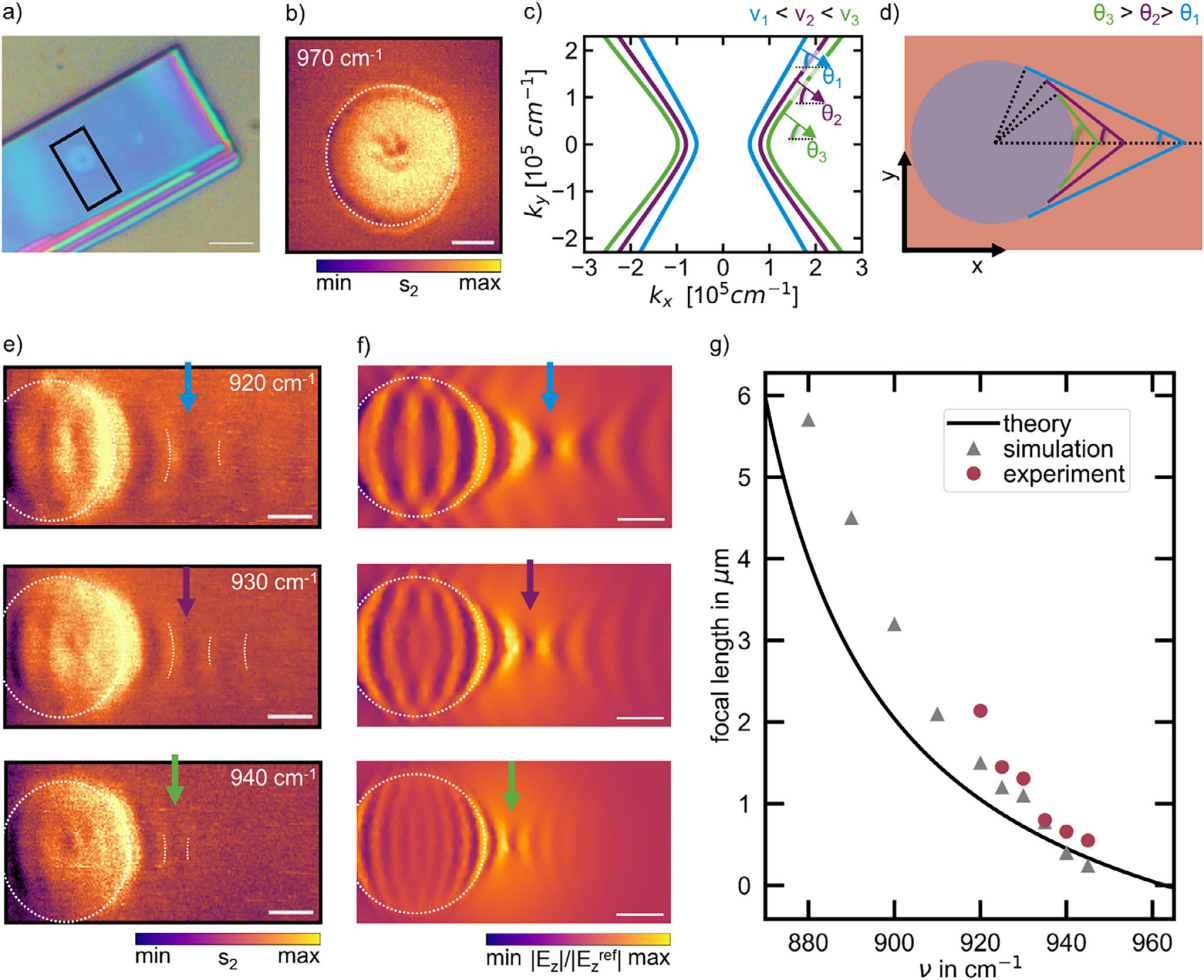
Optically programmed focusing structure. (a) Optical microscope image of an optically programmed circular crystalline launching structure in IST (cIST disk) below α‐MoO_3_ ($d_{\alpha -\text{Mo}O_{3}}$ = 392 nm). The scale bar is 5 µm. (b) s‐SNOM amplitude image of the cIST disk in RB3 of α‐MoO_3_ at 970 cm^−1^. At this frequency, the polaritons propagate under an angle close to zero through the α‐MoO_3,_ providing a direct image, also called a super‐resolution image, of the buried cIST disk. (c) IFC for three different frequencies increasing from 920 (blue) to 930 (purple) to 940 cm^−1^(green). (d) Sketch of the focusing of the cIST disk below the α‐MoO_3_ for the same frequencies as in (c). (e) s‐SNOM amplitude images and (f) electric field simulations of the focusing of α‐MoO_3_’s phonon polaritons by the cIST disk for three exemplary frequencies (same as in (c) and (d)). The scale bars in (b), (e), and (f) are 1 µm. (g) Focal length as a function of incident frequency extracted from s‐SNOM amplitude images (purple dots) and simulations (grey triangles) as in (e) and (f), together with the theoretical behavior (black solid line).

We now optically program a circular cIST launching structure (cIST disk) to focus the polaritons. In the optical image in Figure 3a, the cIST disk is visible as a bright circle. As we program the cIST disk after having deposited α‐MoO_3_, we cannot characterize its exact size and shape by direct measurements (e.g. by atomic force microscopy, as it is done for conventionally fabricated structures). We overcome this issue by performing a super‐resolution s‐SNOM amplitude image (Figure 3b) at 970 cm^−1^, close to the lower bound of the RB3 (956.7 – 1006.9 cm^−1^). At this frequency, the polaritons propagate at an angle close to zero through the slab such that they provide a direct image of the buried structure, a so‐called super‐resolution image [48, 49, 50, 61, 62, 63] (see Note S6). Therefore, we interpret the circular structure of high amplitude as a direct image of the cIST disk below the α‐MoO_3_ flake.

Due to the in‐plane hyperbolicity of α‐MoO_3_ in the RB2, the cIST disk is expected to launch polaritons at any point of its circumference, which are focused to a common point along the *x*‐axis, as sketched in Figure 3d as observed previously for gold launching disks [18, 20, 21], gold antennas [19], and bent silver nanowires [22] on top of α‐MoO_3_. The focal length is determined by the radius of the launching structure and the complementary angle θ =$\;\frac{\pi }{2}-\alpha$ to the opening angle α of the IFC [20]:

(1) $$f=R\;\left(\sqrt{1+\frac{1}{\tan^{2}\theta }}-1\right)=R\left(\sqrt{1-\frac{\varepsilon_{x}\left(v\right)}{\varepsilon_{y}\left(v\right)}}-1\right)$$

The second step uses the relation for the opening angle of the IFC $\tan\left(\alpha \right)=\sqrt{-\frac{\varepsilon_{x}\left(v\right)}{\varepsilon_{y}\left(v\right)}}\;$, which is valid for thin α‐MoO_3_ flakes. Since the opening angle of the IFC decreases with increasing illumination frequency (c.f. Figure 3c), the focal length decreases with increasing illumination frequency, as also reported experimentally [18, 19, 20, 22].

To visualize the focusing of the phonon polaritons in α‐MoO_3_ by the cIST disk and to study the tuning of the focal length experimentally, we show s‐SNOM amplitude images at three different frequencies in the RB2 in Figure 3e. We support our s‐SNOM amplitude images with electric field simulations (c.f. Figure 3f). In both experiment and simulation, we observe curved bright fringes (indicated by white dashed lines) right to the cIST disk (marked by white dashed circumference) that first resemble the curvature of the cIST disk and can be described with a narrowing envelope. We identify the focal point where the curvature of the wave fronts is inverted, and the envelope diverges. We marked the focal point with arrows in experiment and simulation images (c.f. Figure 3e,f). In agreement with the theoretical expectations, with increasing illumination frequency, the focal length decreases, and the fringe spacing corresponding to the polariton wavelength decreases. From s‐SNOM amplitude images and simulations, as shown in Figure 3e,f, we extract the focal length as a function of illumination frequency (c.f. Figure 3g). The experimentally extracted focal length agrees well with that found in our simulation study. However, especially for lower frequencies (longer focal lengths), the focal lengths extracted from experiment and simulation deviate from the theoretical expectation, which relies on the assumption of thin flakes. A similar deviation has been observed in the literature when α‐MoO_3_ flake thicknesses are above 300 nm [20]. Since our flake is 392 nm thick, our findings agree with that in the literature. Our results on phonon polaritons in α‐MoO_3_ focused by an optically programmed cIST disk agree well with those observed by conventionally fabricated metallic focusing structures in the literature [18, 19, 20, 21, 22]. By tuning the illumination frequency, we demonstrate shifting the focal length from 0.55 to 2.14 µm. Our achieved tuning range of the focal length is slightly larger than that achieved by Martín‐Sánchez et al. [18]. (0.6‐1.7 µm) and Zheng et al. [19]. (0.6–1.6 µm) who relied on conventionally fabricated focusing structures. Thereby, we demonstrate that our fast optical programming of cIST disks allows for similar focusing qualities as conventionally fabricated metallic disks while offering much faster turnaround times in fabrication and the opportunity for post‐processing adaptation.

Post‐processing adaptation of the disk radius can be exploited to further tune the focal length. For example, an s‐SNOM image of a cIST disk of smaller radius in Note S7 demonstrates a shorter focal length similar to observations for conventionally fabricated focusing disks [19, 20]. With our fabrication scheme, the disk radius can be changed either by reconfiguring the disk radius or rewriting the disk within 30 s, as we demonstrate in Note S3.

Our concept of optically programming polariton launching structures is applicable to a wide range of thicknesses of vdW materials, as we already demonstrated for hBN flakes with thicknesses ranging from 35 to 465 nm [44]. Thus, the thickness of the slab does not pose a limitation for our optical fabrication process. The thickness strongly impacts the confinement. We expect the confinement can be further increased by choosing thinner α‐MoO_3_ flakes, as the polariton wavelength decreases with decreasing α‐MoO_3_ flake thickness, as we elaborate in Note S8.

**FIGURE 4:**
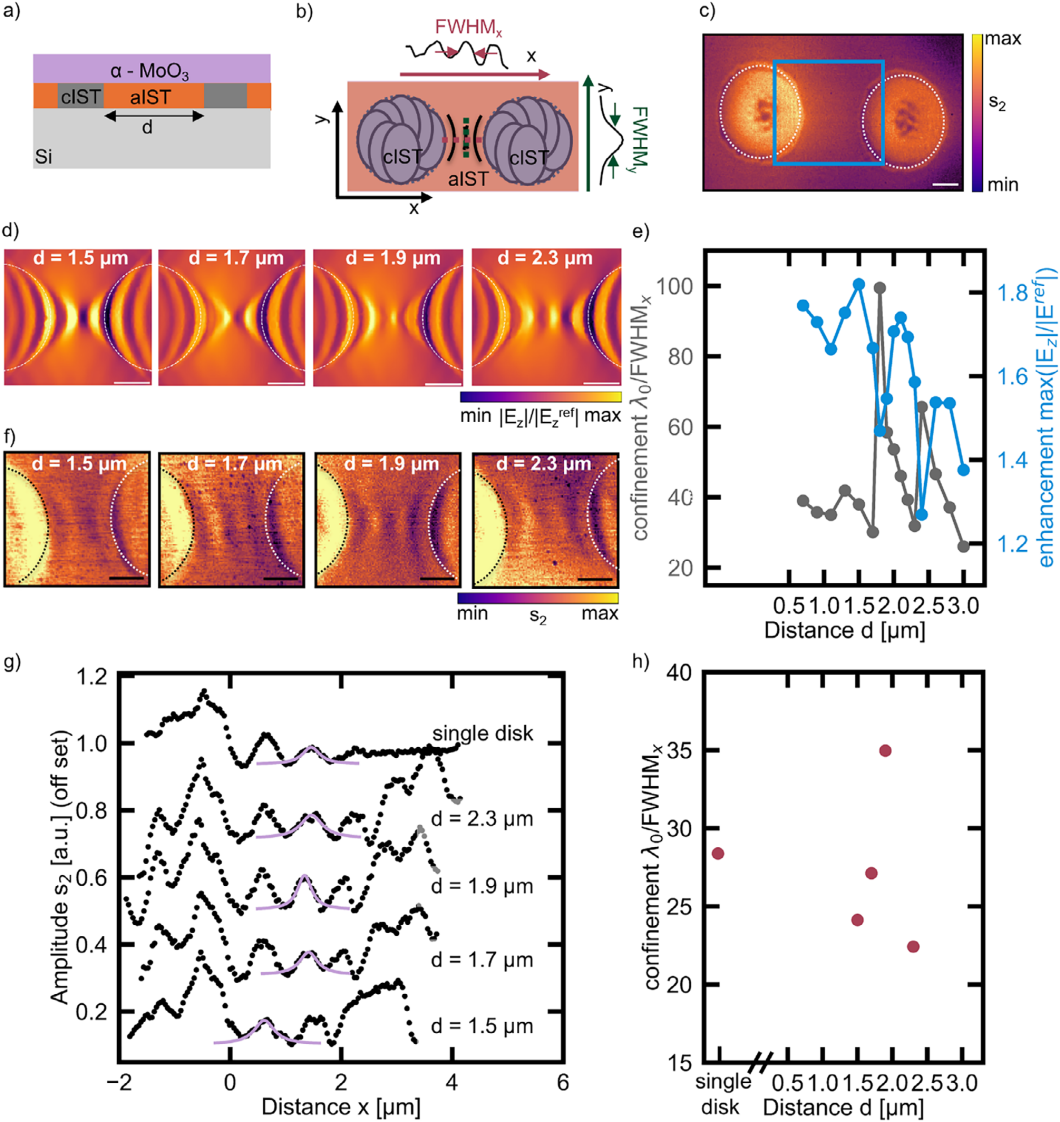
Matching the focal point of two optically programmed focusing structures to confine phonon polaritons in α‐MoO_3_. Sketched (a) side view ($d_{\alpha -\text{Mo}O_{3}}$ = 392 nm) and (b) top view of two crystalline launching structures (double disks) whose focal points overlap. (c) Super‐resolution image of the optically programmed double disks taken at 970 cm^−1^ with s‐SNOM. (d) Simulations and (f) s‐SNOM amplitude images at 930 cm^−1^ of the two focusing disks for four different distances *d* increasing from left to right. The scale bars correspond to 1 µm. (e) Field confinement λ_0_/FWHM_x_ (c.f. inset in (b)) and enhancement along the optical *x*‐axis at the focal point as a function of disk distance d, extracted from simulations such as displayed in (d). (g) Line profiles of a single launching disk (top) extracted from s‐SNOM images in Figure 3e and line profiles of two launching disks whose distance decreases from top to bottom extracted from s‐SNOM images in (f). (h) Confinement λ_0_/FWHM_x_ along the *x*‐direction determined from line profiles extracted from experimental s‐SNOM images (red dots) as a function of disk distance.

Further confinement and enhancement of in‐plane isotropic polaritons is usually achieved by employing nanocavities [64, 65]. For in‐plane anisotropic polaritons, the concept of a cavity becomes more complex due to the lack of rotational symmetry for 2D in‐plane focusing compared to the case of isotropic polaritons. By adding a second cIST disk to the focusing cIST disk discussed in Figure 3 (see sketch in Figure 4a,b) and spatially overlapping the two focal points, we intend to experimentally realize the first step toward a nanocavity for in‐plane hyperbolic polaritons. Our double disk geometry was inspired by Álvarez‐Pérez et al. [23] who theoretically proposed a nanocavity for in‐plane hyperbolic PhP in a geometry of an etched α‐MoO_3_ flake resembling a hyperbolic shape. To the best of our knowledge, this approach has not been followed experimentally in previous literature. Hence, our double disk geometry is the first experimental realization toward a nanocavity for in‐plane hyperbolic polaritons. A detailed comparison of the theoretically proposed nanocavity by Álvarez‐Pérez et al. and our experimentally realized geometry is provided in Note S10. In the following, we study field confinement and enhancement in the double disk geometry.

The confinement along the *x*‐ and *y*‐directions is determined by the full‐width‐at‐half‐maximum (FWHM_x/y_) in the *x*‐ and *y*‐directions of the maximum closest to the middle of the two disks, as sketched in Figure 4b in red and green, respectively. Figure 4c shows a super‐resolution s‐SNOM image taken at 970 cm^−1^ of the two crystalline disks in the amorphous surrounding. We use this super‐resolution image to determine the distance between the disks experimentally to be 1.9 µm.

To prove tailoring the confinement and field enhancement of polaritons, we first perform numerical simulations to tailor the interference of the polaritons by varying the disk distance *d*. Figure 4d shows simulations of the electric field along the *z*‐direction for four exemplary disk distances *d* increasing from left to right from 1.5 to 2.3 µm. The displayed simulations show the area between the two cIST disks marked by the blue rectangle in Figure 4c. Between the two cIST disks that are marked by white dashed circumferences, we observe alternating minima and maxima along the *x*‐direction. By increasing the disk distance, the number of maxima between the two disks increases, and the FWHM of the maxima in *x*‐ and *y*‐directions is tailored. As the confinement of the field λ_0_/FWHM_x/y_ is directly related to FWHM_x/y_ in the *x*‐ and *y*‐directions, respectively, we expect a strong dependence of the confinement on disk distance. To quantify this, we extract line profiles (as sketched by the dashed lines in Figure 4b) from the simulations along *x*‐(red) and *y*‐direction (green). We determine the FWHM of the maximum closest to the middle of the two disks, as indicated in the line profiles sketched in Figure 4b, to calculate the confinement. Further, we determine the field enhancement (max. value of the referenced electric field) from the same maximum. Confinement and field enhancement along the *x*‐direction from simulation are displayed as a function of disk distance in Figure 4e. The field enhancement along the *x*‐direction from simulation is maximal at disk distances of about 1.5 (field enhancement of 1.8) and 2.1 µm (field enhancement of 1.7). These distances correspond to the cases where the polaritons launched by the two disks interfere constructively. The maxima close to the center overlap, leading to a maximal field enhancement (detailed discussion in Note S10). The confinement along the *x*‐direction from the simulation, instead, is maximal at 1.8 µm disk distance (λ_0_/99), close to the geometric overlap of the focal points of the two disks. The maximal confinement along the *y*‐direction of λ_0_/45 we find at a disk distance of 2.4 µm (see analysis in Note S9). Consequently, analyzing the simulations, we find that by tailoring the disk distance we can either maximize field enhancement or confinement.

Exploiting the fast prototyping of our optically programmed double disks, we further corroborate our numerical findings by studying the polariton interference for four different distances by taking s‐SNOM amplitude images (see Figure 4f). The distances correspond to the distances for which we show simulations in Figure 4d. In the s‐SNOM amplitude images, we observe similar patterns as in the simulations. To quantify the confinement in the *x*‐direction, we extract line profiles in the *x*‐direction through the middle of the two disks (c.f. red dashed line in the sketch in Figure 4b) and compare them to a line profile extracted for a single launching disk in Figure 4g. We determine the FWHM_x_ of the maximum closest to the middle (for the single disk closest to the focal point) by fitting a Lorentzian function. The experimental confinement in the *x*‐direction is plotted in Figure 4h. We observe an increased confinement along the *x*‐direction of λ_0_/35 = λ_p_/2.7 at a disk distance of 1.9 µm, in good agreement with the observed maximal confinement in simulations at 1.8 µm. This disk distance corresponds to the distance where the focal points of the left and right disks approximately overlap. Along the *y*‐direction (see analysis in Note S9), we find an enhanced confinement of λ_0_/20 = λ_p_/1.4 at a disk distance of 1.7 µm. Compared to the confinement of the single disk (λ_0_/28 = λ_p_/2.1 along the *x*‐direction and λ_0_/10 = λ_p_/0.8 along the *y*‐direction), the double disk arrangement allows for an increase in the confinement along the *x*‐direction by a factor of 1.25 and along the *y*‐direction by a factor of 2.

Our results on confinement for the single disk arrangement are comparable to those observed in the literature [13, 18, 19, 20, 21], e.g., Martín‐Sánchez et al. [18]. investigated a single gold disk and achieved a confinement of λ_0_/28 along the *x*‐direction and of λ_0_/34 along the y‐direction (c.f. detailed comparative literature study in Note S2). Further tailoring of confinement can be achieved by tuning the illumination frequency (see Note S11).

We envision that confinement and field enhancement could be further enhanced by tailoring the size and distance of the two disks. Moreover, the interference and thus confinement and field enhancement depend on the launching phases [23]. Thus, investigating the influence of the launching phase and the illumination directions is of high interest for future work. Field enhancement and confinement might be even further enhanced when adapting the shape of the launching structures [18] such that they resemble a hyperbolic shape [23] and using thinner flakes, as the confinement generally increases for thinner flakes (c.f. Note S8). A comprehensive discussion of all parameters relevant for achieving optimal performance is provided in Note S10.

As the confinement increases of a factor of 2 for the double disk geometry compared to the single disk, our double disk geometry might already serve as a nanocavity for in‐plane hyperbolic polaritons. Our investigation reveals that the realization of a nanocavity for in‐plane hyperbolic polaritons becomes a complex problem, as the numerous parameters discussed above play a crucial role. With our platform, it will now be possible to quickly explore many other geometries and configurations to elucidate the still open physical question of whether launching and reflecting structures can give rise to resonance modes of in‐plane hyperbolic polaritons.

## 3 Discussion

In this work, we combine the polariton‐hosting vdW material α‐MoO_3_ with the phase‐change material IST, as this allows for fast prototyping of reconfigurable polariton optics. Polariton launching structures can be optically programmed into the IST through the α‐MoO_3_ flake. Our fabrication scheme stands out due to the few fabrication steps needed and thus fast turnaround times, i.e. layer stack fabrication within a few hours and generation of launching structures within 30 s. Moreover, it stands out due to the optical programming of structures after flake deposition and thus flexible alignment of the structure with the flake axes, and due to the opportunity for reconfiguration and addition of structures to already fabricated structures on the very same flake. Hence, it is a versatile platform to steer and study anisotropic polaritons fast and efficiently. We optically program launching stripes through the exfoliated α‐MoO_3_ flake, what allows us to align the launching structures of different orientations with the crystal axis. The aligned launching stripes enable steering the propagation direction of the polaritons and also retrieving the IFC experimentally. We exploit the fast optical programming of crystalline launching disks to focus the polaritons. By varying the illumination frequency, we tailor the focal length from 0.55 to 2.14 µm. At 930 cm^−1^ we find a field confinement of λ_0_/28 = λ_p_/2.1 along the *x*‐direction and λ_0_/10 = λ_p_/0.8 along the *y*‐direction. Both the tuning range of the focal length and the confinement achieved by an optically programmed cIST disk are comparable to values in the literature for conventionally fabricated metal disks, demonstrating the high quality of the optically programmed structures. Finally, we add a second disk to the first focusing disk in a post‐processing step. Thereby, we showcase a first experimental realization toward a nanocavity for the anisotropic polaritons in α‐MoO_3_, which has by now only been proposed theoretically [23]. We add double disks of different disk distances to the very same flake to tailor field confinement and enhancement. We find a maximal field confinement along the x‐direction of λ_0_/35 = λ_p_/2.7 at a disk distance of 1.9 µm and along the y‐direction of λ_0_/20 = λ_p_/1.4 at a disk distance of 1.7 µm. By employing the double disk structure, we can enhance the confinement along the *x*‐direction by a factor of 1.25 and along the *y*‐direction by a factor of 2 compared to the single disk.

Launching structures are needed to characterize the directionality of polaritons in vdW materials. Characterization of highly directional polaritons in other vdW materials such as the in‐plane anisotropic vdW materials β‐Ga_2_O_3_ [66]_,_ α‐V_2_O_5_ [9], or LiV_2_O_5_ [10] can be facilitated by the fast prototyping of polariton optics with IST. We envision the optical programming of polariton optics for vdW materials with IST can also facilitate and fasten the exploration of fundamental optical phenomena on the nanoscale such as high directionality [11, 12, 13], planar refraction [14, 15], focusing [15, 18, 19, 20, 21, 22] and negative reflection [23] and also topological transitions that have been studied with coupled polaritons in vdW heterostructures [13].

The programming scheme is not limited to single nanostructures but, in principle, much more complex structures such as metasurfaces could be optically programmed into the IST similar to metasurfaces programmed into GST to focus polaritons in hBN [33]. These metasurfaces will allow for advanced in‐plane steering of the polaritons [67, 68]. We envision that employing metasurfaces with specific orientations with respect to the crystal axis will also enable highly directional far‐field emission [69]. Hence, our IST platform will facilitate and speed up the realization of advanced nanophotonic devices for highly directional emission and energy transport.

## 4 Methods

### 4.1 Sample Fabrication

Using direct current and radio frequency sputter deposition, a 50 nm (sample 1 in Figure 1, Figure 3 and Figure 4) and 100 nm (sample 2 in Figure 2) thick layer of amorphous In_3_SbTe_2_ and a 20 nm thick capping layer of ZnS: SiO_2_ were applied on a Si substrate. The capping layer (80% ZnS, 20% SiO_2_) prevents the sample from oxidation. α‐MoO_3_ thin slabs were prepared by mechanical exfoliation of bulk α‐MoO_3_ crystals (Alfa Aesar) using Nitto adhesive tape (Nitto Denko Co., SPV 224P). To further reduce the thickness, a second exfoliation step was performed by transferring the single thin slabs from the tape onto a transparent polydimethylsiloxane (PDMS) stamp. The resulting thin slabs were examined under an optical microscope, and homogeneous regions with large lateral dimensions were selected. The thickness of these slabs was determined directly under the optical microscope by the non‐destructive method described in ref [70] to select the most suitable ones. The determined thicknesses were afterward double‐checked by AFM measurements. Finally, these single thin slabs were then transferred onto the previously described amorphous PCM sample using a dry‐transfer technique [71].

### 4.2 Optical Switching

A home‐built laser switching setup was used to crystallize the PCM locally. Here, the light of a 660 nm laser diode is focused on the sample with a 10‐fold objective with NA = 0.25. Each individual crystallized spot for the disk structures was created by applying 100 pulses with a power of 110 mW and a pulse length of 800 ns. Due to the elliptical beam profiles, the crystallized spots are elliptical. One elliptical crystallized spot has a lateral extension of approximately 1 × 1.5 µm^2^. To crystallize the crystalline launching stripes, 21 pulses with a power of 19 mW and a pulse length of 600 ns were employed. The sample was placed on a Thorlabs piezo stage, allowing for precise movements of the sample. The crystalline launching structures are created by placing multiple crystalline spots next to each other. The crystalline disks are created by placing multiple crystalline spots along a ring of the desired radius.

### 4.3 s‐SNOM

In s‐SNOM, a sharp metallic tip is brought into proximity to the sample surface. Upon illumination by a laser, the tip acts as an optical antenna leading to strongly enhanced near‐fields at the tip's apex. The scattered light is detected interferometrically to extract amplitude and phase. We demodulate the signal at the second order of the tip‐oscillation frequency. As s‐SNOM is sensitive to local changes in the permittivity of the sample and to local near‐fields [54], it can directly probe the near‐fields of the polaritons [2, 3, 4, 55, 57]. We used a commercially available s‐SNOM (NeaSNOM neaspec GmbH) to image the polaritons in MoO_3_ on IST. We combined commercially available quantum cascade lasers (MIRcat‐QT Mid‐IR Laser by Daylight Solutions Inc.) with a liquid nitrogen‐cooled Mercury Cadmium Tellurium detector to investigate polaritons in the frequency range from 900 to 945 cm^−1^ and in the frequency range from 960 to 970 cm^−1^ for the super‐resolution regime. We used the pseudoheterodyne detection scheme to obtain s‐SNOM amplitude and phase [53]. We performed our measurements using tapping amplitudes between 60 and 70 nm and demodulated the signal at the second demodulation order.

### 4.4 Numerical Field Simulations

Numerical simulations were performed with the commercial solver CST Studio Suite from Dassault Systèmes. The simulation was modelled with plane wave excitation using p‐polarized light incident at angle (θ, φ) = (60°, 180°). Open boundaries with added free‐space padding where needed were set.

The constant loss‐free refractive index of 3.4 was used for Si. The dielectric function of IST is shown in Figure 1b. A constant refractive index of 2.1 was assumed for the ZnS: SiO_2_. The anisotropic dielectric function of MoO_3_ was modelled according to Figure 1a.

The electric field was extracted 1 nm above the sample surface. The fields were normalized with respect to the incident field. Effects of the SNOM tip were not considered in our simulations.

## Author Contributions

L.J., L.C. and T.T. conceived the project with assistance by P.A.‐G. E.T.‐G., C.L., and A.T.M.‐L. contributed to preliminary sample fabrication and preliminary s‐SNOM measurements. E.T.‐G. performed the final sample fabrication. U.S. and A.M. performed the s‐SNOM measurements. L.J. supervised the s‐SNOM measurements. E.T.‐G. assisted in the s‐SNOM measurements and their interpretation. U.S. performed the numerical field simulations under the supervision of L.C. L.J., U.S., A.M., and L.C. analyzed the data. G.A.‐P. and C.L. provided theoretical assistance and support. P.A.‐G. supervised E.T.‐G., C.L., and A.T.M.‐L. M.W. provided sputtering equipment and phase‐change material expertise. All authors contributed to writing the manuscript.

## Conflicts of Interest

The authors declare no conflicts of interest.

## Code Availability

The computer codes developed for this study are available from the corresponding author upon request.

## Supporting information




**Supporting File 1**: advs77633‐sup‐0001‐SuppMat.pdf.

**Supporting File 2**: advs77633‐sup‐0002‐Movie1.mp4.

## Data Availability

The data that support the findings of this study are available from the corresponding author upon reasonable request.
