# Supplementary material for: Fast Programming of in‐Plane Hyperbolic Phonon Polariton Optics through Van der Waals Crystals Using the Phase‐Change Material In3SbTe2
Source: Adv Sci (Weinh). 2026 Sep 8:e77633. Online ahead of print. doi: 10.1002/advs.77633 (PMC13554493; doi:10.1002/advs.77633)
Supplement: Supplementary file 1 [file ADVS-9999-e77633-s001.pdf]

Supplementary Information

Fast Programming of In-Plane Hyperbolic Phonon Polariton Optics Through van der Waals Crystals using the Phase-Change Material In_3SbTe_2

Author(s) and Corresponding Author(s)*:

Lina Jäckering*,+,[1,2], Umberto Saldarelli+,[1,2], Aaron Moos[1,2], Lukas Conrads[1,2], Enrique Terán-García[3,4], Christian Lanza[3,4], Aitana Tarazaga Martín-Luengo[3,4], Gonzalo Álvarez-Pérez[5], Pablo Alonso-González[3,4], Matthias Wuttig[1,2], Thomas Taubner*,[1,2]

jaeckering@physik.rwth-aachen.de

+both authors contributed equally

Affiliations

[1]1st Institute of Physics (IA), RWTH Aachen University, 52074 Aachen, Germany

[2]Juelich-Aachen Research Alliance (JARA-FIT), 52425 Juelich, Germany

[3]Department of Physics, University of Oviedo, 33006 Oviedo, Spain

[4]Center of Research on Nanomaterials and Nanotechnology, CINN (CSIC-Universidad de Oviedo), 33940 El Entrego, Spain

[5]Istituto Italiano di Tecnologia, Center for Biomolecular Nanotechnologies, Via Barsanti 14, 73010 Arnesano, Italy.

This PDF file includes:

Supplementary Note 1: Comparison of our fast fabrication of polariton optics to conventional fabrication schemes

Supplementary Note 2: Comparative study of reconfigurable polariton optics and focusing structures

Supplementary Note 3: Optical programming and reconfiguring of polariton optics

Supplementary Note 4: In-plane hyperbolic polaritons

Supplementary Note 5: Polariton wavevector from s-SNOM line profiles

Supplementary Note 6: Super-resolution imaging

Supplementary Note 7: Tuning the focal length by changing the disk radius

Supplementary Note 8: Thickness dependence of PhPs in α-MoO_3 on IST

Supplementary Note 9: Enhancement and confinement along optical y-axis

Supplementary Note 10: Tailoring confinement and field enhancement by disk distance variation

Supplementary Note 11: Tailoring confinement and field enhancement by varying illumination frequency

Supplementary Note 12: Derivation of the dispersion relation of highly confined PhPs in the investigated layer stack

Supplementary Note 1: Comparison of our fast fabrication of polariton optics to conventional fabrication schemes

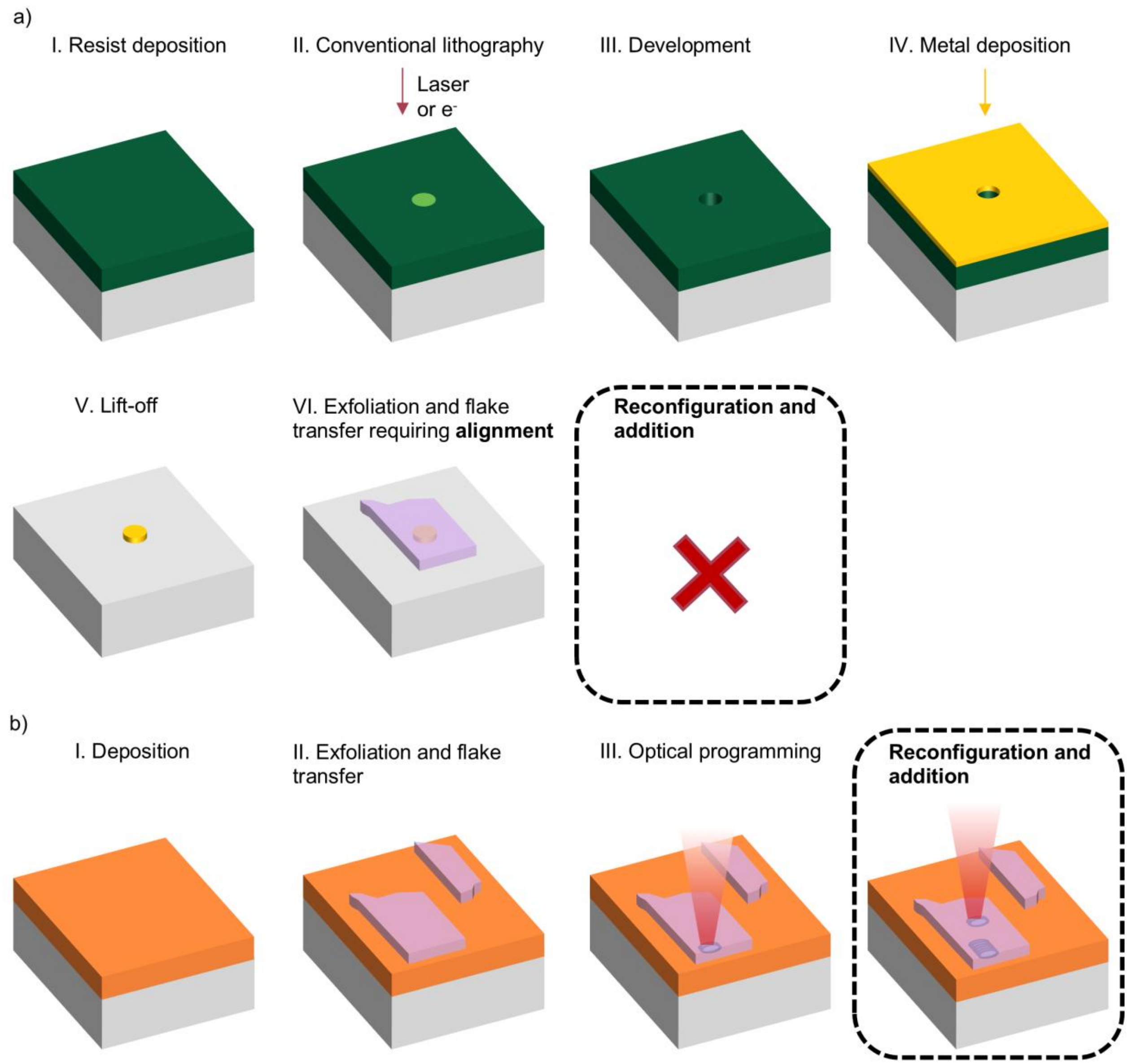

Figure S1: Comparison of our fast fabrication of polariton optics to conventional fabrication schemes. *a) Conventional fabrication scheme of metallic polariton optics e.g. launching disks. Conventionally, metallic disks are fabricated via electron beam or optical lithography that involve the subsequent fabrication steps: (I) a resist mask is deposited on the substrate that is subsequently (II) patterned using an electron beam or a laser. Afterwards (III) the resist is developed and (IV) the metal is deposited. (V) In the lift-off the remaining resist with the deposited metal on top is removed leaving the aimed metal disk. To deposit an α-MoO_3*

flake, first the flakes are exfoliated mechanically onto a different substrate. Then, the flake of choice is transferred and aligned onto the previously fabricated metal disk. (VI) Afterwards, there is no option for adding another metal structure to the same flake nor an option for reconfiguration of the fabricated structure. b) Our fast prototyping scheme is based on only three steps: First, (I) the amorphous IST film is deposited onto the substrate. Then, (II) α-MoO_3 flakes are exfoliated and transferred onto the amorphous IST. Finally, (III) a α-MoO_3 flake is chosen and the desired launching structures optically programmed through the α-MoO_3 flake. Thus, the desired structure can be aligned to the crystal axes flexibly. Our fabrication scheme allows us to add further launching structures to the same flake and also to reconfigure the previously fabricated structure by further crystallization.

Conventionally, the fabrication of polariton launching structures relies on multi-process fabrication schemes such as electron beam or optical lithography[1]. To contrast our fast three step fabrication scheme with conventionally used, time-consuming lithography, we shortly outline the process steps for both fabrication schemes in Figure S1. In conventional lithography[1] (c.f. Figure S1a) the fabrication process starts with the deposition of a resist mask onto the substrate (I). Next, the resist is patterned using an electron beam or laser (II). Afterwards, the resist is developed (III) and the metal deposited (IV). In a subsequent lift-off the remaining resist together with the metal on top of it is removed leaving the aimed metal launching structure (V). In order to align an α-MoO_3 flake with the fabricated metal structure, first, α-MoO_3 flakes are mechanically exfoliated onto a different substrate. Then, an α-MoO_3 flake is chosen, transferred and aligned with the metal structure (VI). Relying on these conventional techniques does not allow for adding another structure to the same flake nor for reconfiguration of the structure.

Our fabrication scheme is based on only three steps (c.f. Figure S1b). First, amorphous IST is deposited onto the substrate, followed by sputtering of a capping layer of ZnS:SiO_2 to prevent the IST from oxidation (omitted in sketches) (I). Secondly, α-MoO_3 flakes are mechanically exfoliated onto a polydimethylsiloxane (PDMS) stamp and transferred onto the thin IST film (III). Thirdly, an α-MoO_3 flake is chosen, and the desired launching structure is optically programmed through the α-MoO_3 by locally crystallizing the IST with a red laser (IV). Our fabrication scheme

allows for adding another launching structure to the same flake and also for reconfiguration of the previously written structure.

To highlight the short turnaround time of our fabrication scheme compared to conventional fabrication schemes of lithography, in the following the overall time is estimated for both fabrication schemes: In conventional lithography, the deposition and patterning of the resist is estimated to several hours, followed by approximately 0.5 to 1 hours for the development, 1 to 2 hours for metal deposition and 0.5 hours for the lift off. Additionally, the exfoliation and subsequent transfer and alignment of an α-MoO_3 flake to the structure takes several hours. These different process steps require different machines that are often located in different facilities. Taking into account the availabilities of the machines, often several days are required for fabrication.

In contrast, the overall time of our fabrication scheme can be estimated to be 1 to 2 hours for deposition of the IST film, 1 hour for mechanical exfoliation of the α-MoO_3 flakes and 30 seconds for optical programming of the launching structures. Using our fabrication scheme, optical structures can be fabricated within a few hours. Moreover, additional structures of varied size and shape for the same flake or different flakes on the same chip can be fabricated within another 30 seconds as the first two steps are not required. Further, size and shape can be adapted in a post-processing step within 30 seconds, in case the desired performance was not achieved with the initial structure. Hence, the use of fewer fabrication steps leads to faster turnaround times.

Supplementary Note 2: Comparative study of reconfigurable polariton optics and focusing structures

Publication	Polariton-hosting material	Tuning mechanism	Free propagation	Nanostructures	Focusing structure	
					tuning range focal point	Confinement
This work	α-MoO_3	Phase change in IST<br>- Non-volatile<br>- Reconfigurable<br>- Modification of size and shape	**Launching** at phase boundary	**Launching and focusing** structure<br><br>**Double disk**	0.55-2.14 µm (frequency variation)	x: $\lambda_0/28$; $\lambda_p/2.1$<br>y: $\lambda_0/10$; $\lambda_p/0.8$<br><br>x: $\lambda_0/35$; $\lambda_p/2.7$<br>y: $\lambda_0/20$; $\lambda_p/1.37$
Jäckering et al.[2]	hBN	Phase change in IST<br>- Non-volatile<br>- Reconfigurable<br>- Modification of size and shape	**Launching** at phase boundary	Circular **resonator**<br><br>**Launching and focusing** structure	1.3-3.4 µm (curvature tuning)	
Lu et al. [3]	$h^{10}BN$<br><br>α-MoO_3	Phase change in IST<br>- Non-volatile<br>- Reconfigurable	Free propagation of HPhP in hBN and α-MoO3 tailored by IST phases	**launching and focusing** antenna for HPhP in α-MoO3	~1-2.8 µm (extracted from Figure 4F) (curvature + frequency tuning)	-
Aghamiri et al. [4]	$h^{10}BN$<br><br>α-MoO_3	Tune charge carrier concentration in $SmNiO_3$ (SNO) via<br>- strong local fields: non-volatile<br>- hydrogenation: non-volatile, global<br>- temperature: volatile, global	Free propagation of HPhP in hBN and α-MoO_3 tailored by tuned local conductivity in SNO;	Triangular **cavity** for HPhP in hBN (not reconfigured nor tailored)<br>Circular, rectangular **resonator** for HPhP in α-MoO_3 (not reconfigured nor tailored)	-	-
Qu et al.[5]	α-MoO_3	α-MoO_3 on graphene: Tuning of fermi energy in graphene volatile		**Launching and focusing** structure	~0.7-7.4 µm) (tuning by disk diameter variation, frequency tuning)<br>~2.8-4.6 µm (extracted from Figure 4f) (by tuning fermi energy in graphene	x: -<br>y: $\lambda_0/33$
Hu et al. [6]	α-MoO_3	α-MoO_3 on graphene: Tuning		**Launching and focusing** structure	-	x: -<br>y: $\lambda_0/21$;

		of fermi energy in graphene volatile				
Liang et al. [7]	α-MoO_3	α-MoO_3 Te-thin film: variation of Te-film thickness		**Launching and focusing** structure	2.29-5.61 μm	x: -<br>y: $\lambda_0/20$; $\lambda_p/1.8$ (λ_p extracted from Figure 4b)
Martín-Sánchez et al. [8]	α-MoO_3	No tuning		**Launching and focusing** structure<br><br>Improved shape	0.6-1.7 μm (tuning frequency)	x: $\lambda_0/28$<br>y: $\lambda_0/34$; $\lambda_p/2$<br><br>x:<br>y: $\lambda_0/50$; $\lambda_p/4.5$
Zheng et al. [9]	α-MoO_3	No tuning		**Launching and focusing** structure	0.6-1.6 μm (extracted from Figure 4G)	x: $\lambda_0/32$<br>y: $\lambda_0/30$; $\lambda_p/1.04$ (λ_p extracted from Figure 4G)

Table S1: Comparative study of reconfigurable polariton optics and focusing structures

A comparative study of reconfigurable polariton optics for polaritons in α-MoO_3 and polariton focusing structures in related literature is summarized in Table S1. We highlight the reconfigurability and compare the tuning range and achieved confinement of focusing structures.

Comparison with previously investigated reconfigurable or tunable polariton optics

Tunable and reconfigurable optics for polaritons in α-MoO_3 have been previously achieved by substrate-engineering, i.e. the substrate of the α-MoO_3 is changed and thereby the polariton propagation is altered. Liang et al.[7] combined α-MoO_3 with thin-films of Te of different thicknesses. Relying on conventional lithography techniques, a focusing gold disk was added, and by fabricating samples of different Te-film thicknesses the focal length was tuned. However, this approach still requires conventional fabrication techniques and film thicknesses cannot be changed afterwards, thus this approach is not suitable for fast prototyping nor reconfiguring of polariton optics. Tunable focusing structures for polaritons in α-MoO_3 have been realized by combing α-MoO_3 with graphene.[5,6] Graphene's optical properties are gate tunable and therefore

also the polariton propagation in the α-MoO_3 above can be tailored upon gating. Thereby, Hu et al.[6] and Qu et al.[5] realized gate-tunable focusing with conventionally fabricated gold-launching structures. Fast prototyping of polariton optics such as circular and rectangular resonators before α-MoO_3 flake deposition has been achieved by Aghamiri et al.[4] by combing α-MoO_3 with the oxide SNO. The conductivity and thus also the optical properties of SNO can locally be tuned by applying high electric fields and thus polariton optics can directly be imprinted. Recently, Lu et al.[3] combined α-MoO_3 with the PCM IST and demonstrated tailoring of the polariton propagation by the two IST phases. Further, they transferred an α-MoO_3 flake onto crystalline antennas of different widths programmed into the IST before. These crystalline antennas focus the polaritons. However, programming of polariton optics after flake deposition and their reconfiguration have not been demonstrated using SNO nor IST. In recent work by our group [2], fast prototyping and reconfiguring of a focusing arc structure programmed for in-plane isotropic phonon polaritons in hBN into IST has been demonstrated. In this work, we follow-up on the previous approach and combine α-MoO_3 with IST to tailor the in-plane hyperbolic phonon polaritons in α-MoO_3. We optically program launching structures through α-MoO_3 into the IST. As we can optically program the structures after flake deposition, we demonstrate a fast prototyping scheme. Furthermore, we show reconfiguration of already fabricated structures.

Comparison with previously investigated static polariton focusing structures

In our work, we tune the focal length of a single focusing structure from 0.55 to 2.14 μm by variation of the illumination frequency and show a confinement of $\lambda_0/28 = \lambda_p/2.1$ along the optical x-axis and of $\lambda_0/10 = \lambda_p/0.8$ along the optical y-axis. By adding a second focusing disk we increase the confinement to $\lambda_0/35 = \lambda_p/2.7$ along the optical x-axis and to $\lambda_0/20 = \lambda_p/1.37$ along the optical y-axis. Our achieved tuning range of the focal length (0.55 to 2.14 μm) is slightly larger than that achieved by Martín-Sánchez et al. [8] (0.6-1.7 μm) and Zheng et al.[9] (0.6-1.6 μm)

who relied on conventionally fabricated focusing structures. The conventionally fabricated focusing structures of different radii by Qu et al.[5] allowed for a slightly larger tuning range of 0.7 to 7.4 μm. The confinement of $\lambda_0/28 = \lambda_p/2.1$ along the optical x-axis and of $\lambda_0/10 = \lambda_p/0.8$ along the optical y-axis achieved with our focusing structures is comparable to that observed for conventionally fabricated focusing structures by Zheng et al. [9] ($\lambda_0/32$ along the optical x-axis and $\lambda_0/30 = \lambda_p/1.04$ along the optical y-axis) and slightly lower than that by Qu et al.[5] ($\lambda_0/33$ along the optical y-axis) and by Martín-Sánchez et al. [8] ($\lambda_0/28$ along the optical x-axis and $\lambda_0/34 = \lambda_p/2$ along the optical y-axis). However, the confinement achieved with the double disk structure ($\lambda_0/35 = \lambda_p/2.7$ along the optical x-axis and $\lambda_0/20 = \lambda_p/1.37$ along the optical y-axis) compares well with the higher values observed in literature. Only the focusing structure optimized to increase the contribution of high wavevectors and decrease that of low wavevector by Martín-Sánchez et al.[8] shows a higher confinement ($\lambda_0/50 = \lambda_p/4.5$ along the optical y-axis). In summary, our work presents fast-prototyping of reconfigurable focusing structures for polaritons in α-MoO_3 that do show a performance in terms of tuning range of the focal length and confinement comparable to previously investigated, conventionally fabricated focusing structures.

Supplementary Note 3: Optical programming and reconfiguring of polariton optics

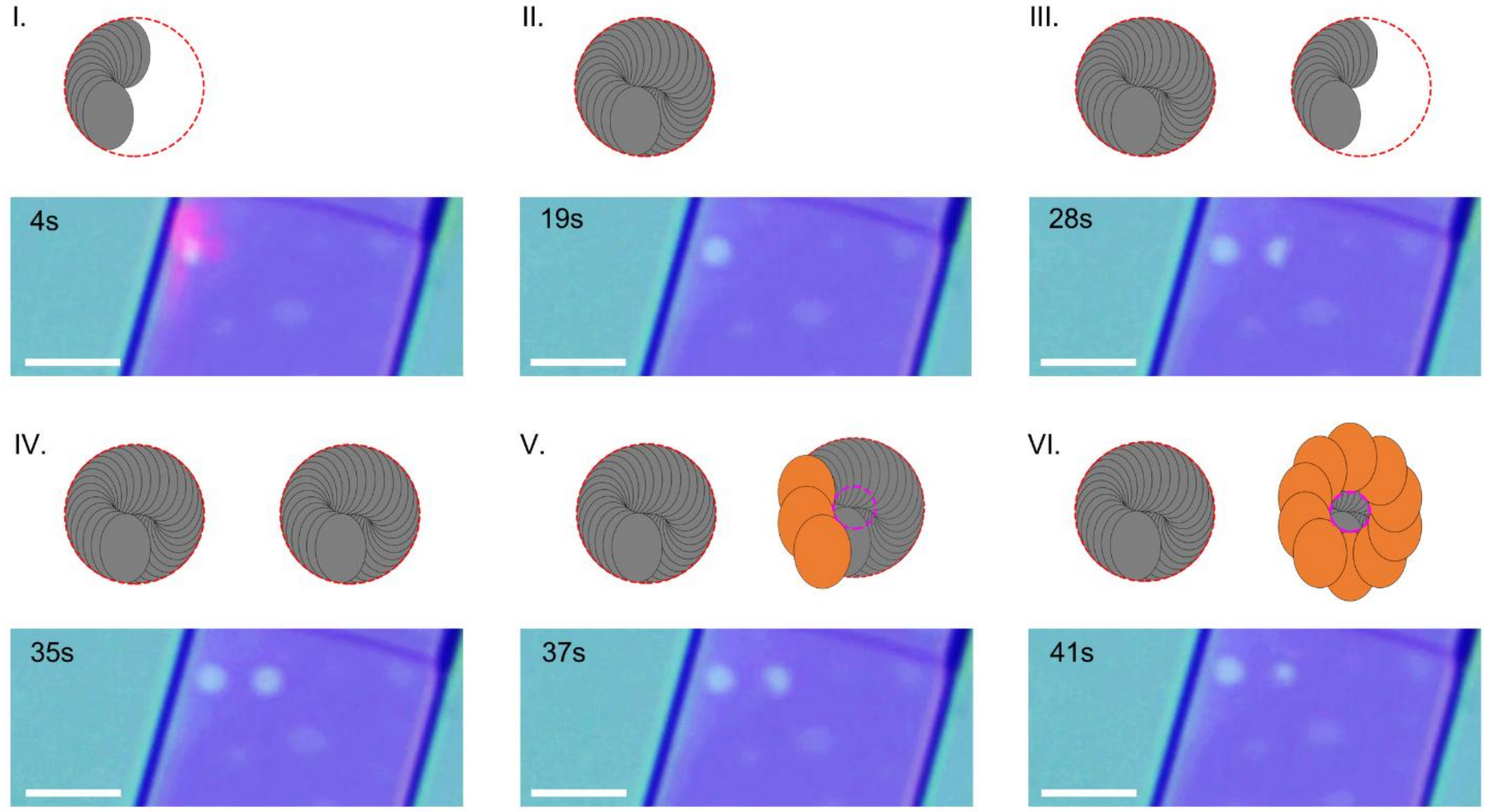

Figure S2: Optical programming and reconfiguring of polariton optics. *Snap shots taken from Supplementary Movie 1 and schematics to guide the reader during the process. In the snap shots, the α-MoO_3 flake (right) appears dark purple on the light blue aIST (left). In the schematics, grey ellipses represent individual crystallization pulses, whereas orange ellipses represent reamorphization pulses. I.-II. Snap shots of the optical programming of a cIST disk by visible laser pulses (red). The cIST disk is visible as bright circle. III.-IV. Optical programming of a second cIST disk. V.-VI. Reconfiguring the second cIST disk to a smaller size by laser pulses for reamorphization. Scale bars are 10 µm.*

Our IST platform enables fast optical programming and reversible reconfiguration of polariton optics. As discussed in the main text, visible laser pulses can be used to reversibly switch IST between its amorphous and crystalline phases directly through the α-MoO_3 flake. This allows for post processing adaptations, i.e. changing shape and size by additional crystallization or by reamorphization.

Supplementary Movie 1 shows live demonstration of the optical programming and optical reconfiguration. In Figure S2, we show snap shots of the movie, together with schematics to guide the reader through the optical programming and reconfiguration process. In the

schematics, grey ellipses represent individual crystallization pulses, whereas orange ellipses represent reamorphization pulses.

We apply elliptical laser pulses (I.) with a power of 42.1 mW and a duration of 800 ns to optically program a cIST disk through the α-MoO_3 flake. After 19 seconds (II.), the optical programming is finished and the cIST disk is visible as a bright circular region.

Afterwards, we add a second cIST disk to the very same flake by additional crystallization with the same parameters (III.). After another 16 seconds (IV.) we have reconfigured the single disk structure to a double disk structure.

Finally, we optically reconfigure the radius of the second cIST disk. We apply laser pulses (V.) with a power of 356.59 mW and a duration of 25 ns to erase parts of the second cIST disk by reamorphization in order to decrease the cIST disk radius. After further 6 seconds (VI.), we have optically reconfigured the radius of the second cIST disk.

Within 41 seconds we demonstrate optical programming of a cIST disk, addition of a second cIST disk and reconfiguration of the second cIST disks radius. Thereby, we clearly demonstrate the fast turnaround times of our fabrication scheme as well as its opportunity for post-processing adaptation.

Supplementary Note 4: In-plane hyperbolic polaritons

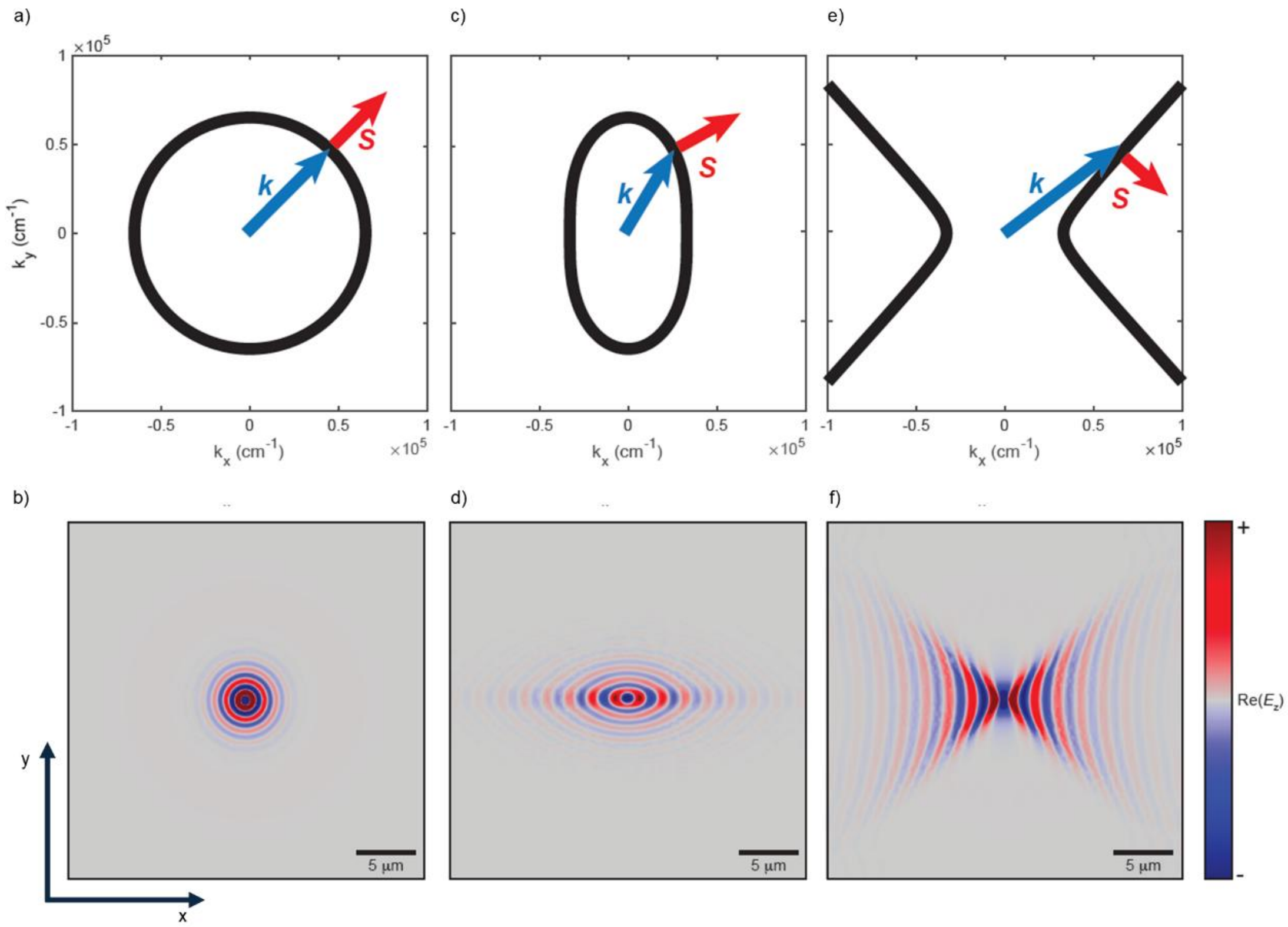

Figure S3: In-plane Hyperbolic Polaritons. *a) IFC in Fourier space (k_x,k_y) at fixed k_z and b) sketch of the in-plane propagation of in-plane isotropic polaritons. c) IFC in Fourier space (k_x,k_y) and d) sketch of the in-plane propagation of in-plane elliptical polaritons. e) IFC in Fourier space (k_x,k_y) and f) sketch of the in-plane propagation of in-plane hyperbolic polaritons. The red-dashed line and the red solid line indicate the polariton wavevector and the Poynting vector, respectively.*

In the case of in-plane isotropic media, the permittivity tensor components along the optical x- and y-axes have the same value. Thus, the isofrequency contour (IFC) which can directly be retrieved from the dispersion equation resembles a circle in the Fourier space (k_x,k_y) at fixed k_z as sketched in Figure S3a.[10,11] As the Poynting vector is parallel to the normal of the IFC, both the Poynting vector and the wavevector are collinear for in-plane isotropic materials as can be observed in Figure S3a.[10,11] The circular IFC leads to an in-plane isotropic propagation of polaritons as sketched in Figure S3b.[10,11]

In the case of in-plane elliptical media, the permittivity along the optical x- and y-axes is different but of the same sign. Thus, the IFC resembles an ellipse (c.f. Figure S3c) and the Poynting vector and the wavevector are not collinear in general.[11] The elliptical IFC leads to an in-plane anisotropic propagation in real space (c.f. Figures S3d).[11]

In the case of in-plane hyperbolic media, the permittivity along the optical x- and y-axes is different and also opposite in sign. This leads to an in-plane hyperbolic IFC.[10–12] Figure S3e sketches the hyperbolic IFC in Fourier space (k_x,k_y) for the case of a negative permittivity along the optical x-axis. From the sketch, we can see that both the Poynting vector and the wavevector are not collinear.[10,11] Furthermore, the IFC restricts the direction of wavevectors. Only wavevectors with an angle γ with respect to the optical x-axis smaller than the IFC opening angle α are allowed.[10,11] This leads to the highly directional propagation polaritons in in-plane hyperbolic media as sketched in Figure S3f.[10,13]

Supplementary Note 5: Polariton wavevector from s-SNOM line profiles

For a quantification of the polariton wavevector in Figure 1 and Figure 2 in the main text, we fitted line profiles extracted from s-SNOM amplitude images as described in the following. In s-SNOM there are typically two launching mechanisms of freely propagating polaritons: tip-launching and edge-launching. The tip-launched polaritons are launched by the sharp s-SNOM tip and reflected, e.g. at a sample edge[14–16], leading to an interference pattern with a fringe spacing of $\delta = \frac{\lambda_p}{2} = \frac{\Pi}{k_p}$.[14–17] The edge-launched polaritons are launched at a discontinuity, such as an edge, a phase-boundary or a defect and interfere with the incident light[17,18]. The resulting interference pattern shows a fringe spacing of $\delta \approx \lambda_p = \frac{2\Pi}{k_p}$ when $k_p >> k_0$[17,18]. In our experiments, we do only observe contributions of polaritons launched at the phase-boundary of IST (edge-launched). We use the following fit function for edge-launched polaritons, considering the geometric decay of $1/x^a$[17], where a has been found to be $a \sim 1$ in previous studies[17], and a linear background in Figure 1, (in Figure 2: C=0):

$$s \sim |A \frac{e^{ik_p(x-x_0)}}{x - x_0} + B + Cx|$$

Here, A, B, C, and x_0 are free parameters.

Supplementary Note 6: Super-resolution imaging

As we program the cIST structures after having exfoliated α-MoO_3 we cannot characterize their exact size and shape by direct measures (e.g. by atomic force microscopy as it is done for conventionally fabricated structures). We overcome this issue by performing a super-resolution s-SNOM amplitude image by tuning our laser source to 970 cm^{-1}, close to the lower bound of the RB3 (956.7 – 1006.9 cm^{-1}) of α-MoO_3. The super-resolution image of the cIST disk is shown in Figure 3b in the main text. We observe a circular area of high amplitude (surrounded by a white dashed contour-line) in the center of the image. Outside the circular area the amplitude is approximately constant and lower. In the upper reststrahlenband both in-plane permittivities are positive ($\varepsilon_x > 0$, $\varepsilon_y > 0$) whereas the out-of-plane permittivity is negative ($\varepsilon_z < 0$).[13] Thus, the propagation behavior is similar to that observed for HPhPs in hBN.[19,20] The hyperbolic PhPs in α-MoO_3 propagate under a restricted angle through the volume of the α-MoO_3 slab. At the lower bound of the upper reststrahlenband, the PhPs in α-MoO_3 will propagate under an angle close to zero, allowing for a direct image of the structure below, the so-called super-resolution imaging. The super-resolution imaging is similar to that observed in hBN, where super-resolution imaging was exploited to visualize gold-disks[19–21] and defects in buried few-layer graphene flakes[22,23]. Previously, canalization based super-resolution imaging of buried gold disks with a α-MoO_3 SiC heterostructure has been demonstrated[24]. Therefore, we interpret the circular structure of high amplitude as a direct image of the cIST disk below the α-MoO_3 flake. In the middle of the bright circle in Figure 3b, we observe a feature having a lower amplitude. The feature of low amplitude in the center aligns with the dark spot visible in the center of the bright crystalline area in the light microscope image (Figure 3a). Most likely, this feature corresponds to a slightly burnt spot during optical programming. We use this direct image to extract size, shape and position of the cIST disk. The crystallized circle has a slightly elongated axis along the y-direction.

Supplementary Note 7: Tuning the focal length by changing the disk radius

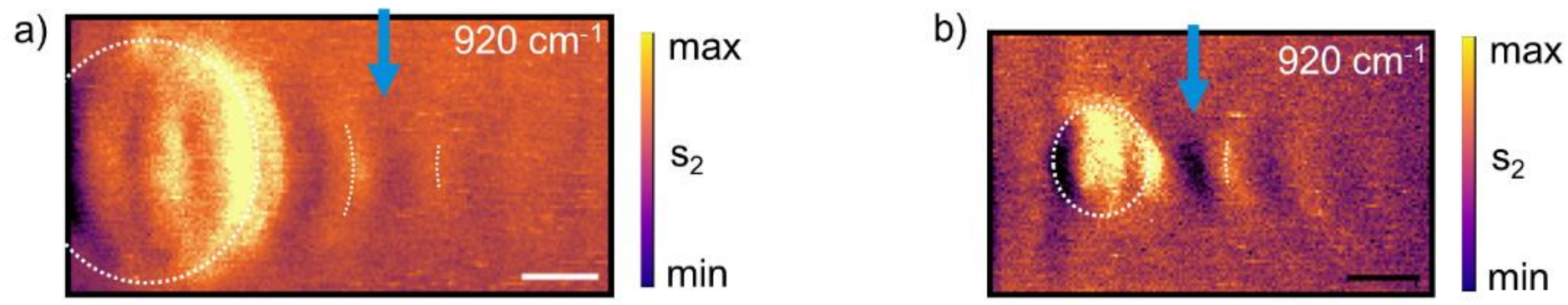

***Figure S4: Tuning the focal length by changing the disk radius**. S-SNOM amplitude images at 920 cm^{-1} of the focusing disk of a) a radius of 1.5 µm discussed in Figure 3 and b) a radius of 0.8 µm on the same flake. The blue arrows indicate the focal length that is decreased for the smaller disk. The scale bars are 1 µm.*

To demonstrate further tuning of the focal length we optically program a cIST disk with a smaller radius R of 0.8 µm and compare its focusing performance to the cIST disk in Figure 3. As the focal length is proportional to the disk radius, we expect the focal length to decrease by decreasing the cIST disk as demonstrated experimentally for conventionally fabricated focusing disks[5,9]. The s-SNOM amplitude images in Figure S4 show that for the same flake and at the same illumination frequency the focal length is decreased for the smaller cIST disk as the blue arrows indicate. As we demonstrate in Supplementary Note 3, the cIST disk radius can be tailored in post-processing on demand. By reconfiguring the disk radius or by rewriting the disk with a different radius, our platform allows for tailoring the focal length on demand within 30 seconds.

Supplementary Note 8: Thickness dependence of PhPs in α-MoO_3 on IST

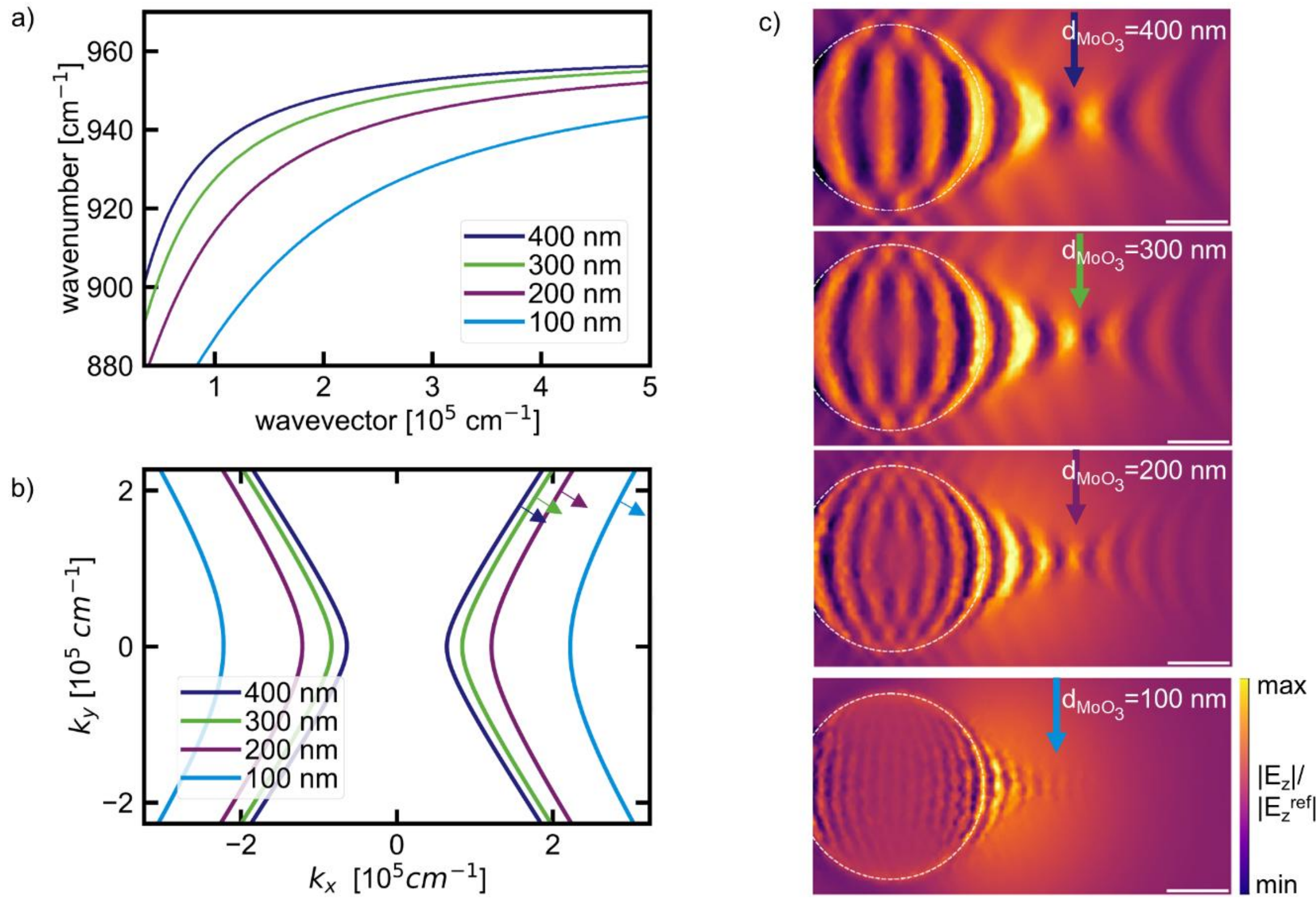

***Figure S5: Thickness Dependence of PhPs in α-MoO₃ on IST**. a) Dispersion of PhPs in α-MoO₃ of different thicknesses on top of amorphous IST in RB2. b) IFC of PhPs in α-MoO₃ of the same thicknesses as in a) on top of amorphous IST at 920 cm⁻¹. c) Simulations of PhPs in α-MoO₃ focused by a cIST disk for different α-MoO₃ thicknesses at 920 cm⁻¹. Scale bars are 1 µm.*

The PhPs in α-MoO_3 are volume-confined modes and out-of-plane hyperbolic in all RBs, hence their dispersion is strongly thickness dependent (c.f. Equation S24 in Supplementary Note 12)[13,25–27]. Figure S5a depicts the dispersion of PhPs in α-MoO_3 of different thicknesses on top of amorphous IST in RB2 and reveals a shift of the dispersion to higher wavevectors for decreasing α-MoO_3 thickness in agreement with previous studies on the thickness dependence[13,25–27]. The thickness-dependent shift of the PhP dispersion implies a higher confinement of the PhPs for thinner α-MoO_3. Consequently, higher confinement with our platform could be achieved by using thinner α-MoO_3 flakes. The propagation length is determined by the phonon lifetime and the confinement and therefore expected to depend on the α-MoO_3 thickness[28].

The focal length is given by the opening angle of the IFC as discussed in Figure 3 in the main text and independent of the α-MoO_3 thickness. Figure S5b shows the IFC of the PhPs in the α-MoO_3 IST platform for different α-MoO_3 thicknesses at 920 cm^{-1}. In the limit of high wavevectors, we do observe very similar opening angles of the IFCs independent of the α-MoO_3 thickness. Hence, we do not expect the α-MoO_3 thickness to influence the focal length. In previous literature, deviations from the theoretical focal length and the experimental have been found for α-MoO_3 thicknesses above 300 nm, raising the open question of the thickness dependence for very thick flakes[5].

Moreover, we perform simulations at 920 cm^{-1} of PhPs focused by a cIST disk for different α-MoO_3 thicknesses (c.f. Figure S5c). In agreement with the IFCs, the simulations reveal similar focal length for α-MoO_3 thicknesses of 400, 300 and 200 nm. For 100 nm α-MoO_3 thickness, the polaritons decay within a shorter length scale as the polariton wavelength is much shorter. Hence, the focal point is hard to identify but seems to be at shorter distances than for the other thicknesses. This deviation might be due to fewer contributions from high wavevectors at this frequency.

Supplementary Note 9: Enhancement and confinement along optical y-axis

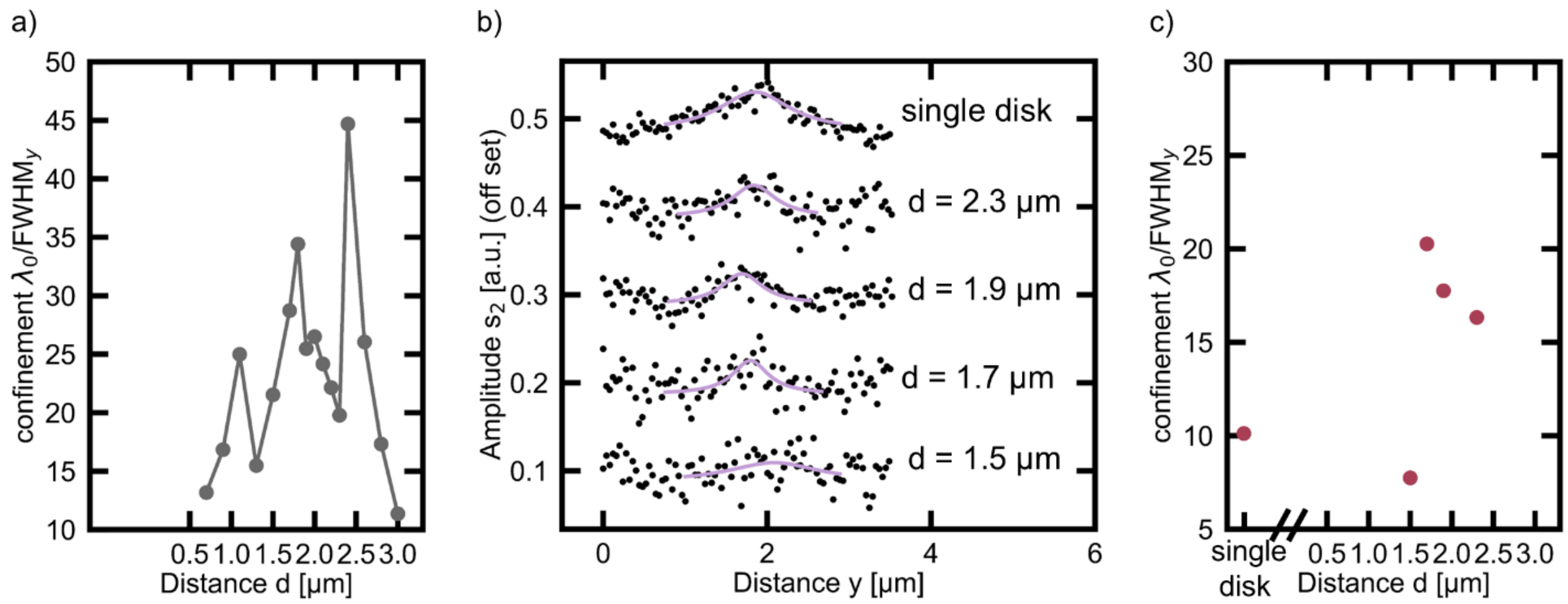

***Figure S6: Enhancement and confinement along optical y-axis**. a) Field confinement $\lambda_0/FWHM_y$ along the optical y-axis as function of disk distance extracted from simulations such as displayed in Figure 4d) in the main text. b) Line profiles of a single launching disk (top) extracted from s-SNOM images in Figure 3d) in main text and line profiles of two launching disks whose distance decreases from top to bottom extracted from s-SNOM images in Figure 4f) in main text. c) Confinement $\lambda_0/FWHM_y$ along y-direction determined from line profiles extracted from experiment (red dots) as a function of disk distance.*

To quantify the influence of the disk variation on the confinement along the y-direction analogous to the x-direction discussed in Figure 4, we extract line profiles from the simulations (c.f. green dashed line in sketch in Figure 4b in main text) and determine the $FWHM_y$ of the maximum closest to the middle of the two disks to calculate the confinement $\lambda_0/FWHM_y$. Confinement along the y-direction is displayed as function of the disk distance extracted from simulations in Figure S6a. The maximal confinement along the y-direction corresponds to $\lambda_0/45$ and is found at a disk distance of 2.4 µm.

To experimentally quantify the confinement along the y-direction, we extract line profiles along y-direction from the s-SNOM amplitude images displayed in Figure 4f through the maximum closest to the middle of the two disks (c.f. green dashed line in Figure 4b in main text; for the single disk closest to the focal point) and compare them to a line profile extracted for a single launching disk in Figure S6b. We determine the $FWHM_y$ of the maximum by fitting a Lorentzian

function. The experimental confinement along the y-direction is plotted in Figure S6c. Along the y-direction we find an enhanced confinement of $\lambda_0/20 = \lambda_p/1.4$ at a disk distance of 1.7 μm. Compared to the confinement of the single disk along the same axis ($\lambda_0/10 = \lambda_p/0.8$) the double disk arrangement allows for an increase in the confinement by factor of 2.

Supplementary Note 10: Tailoring confinement and field enhancement by disk distance variation

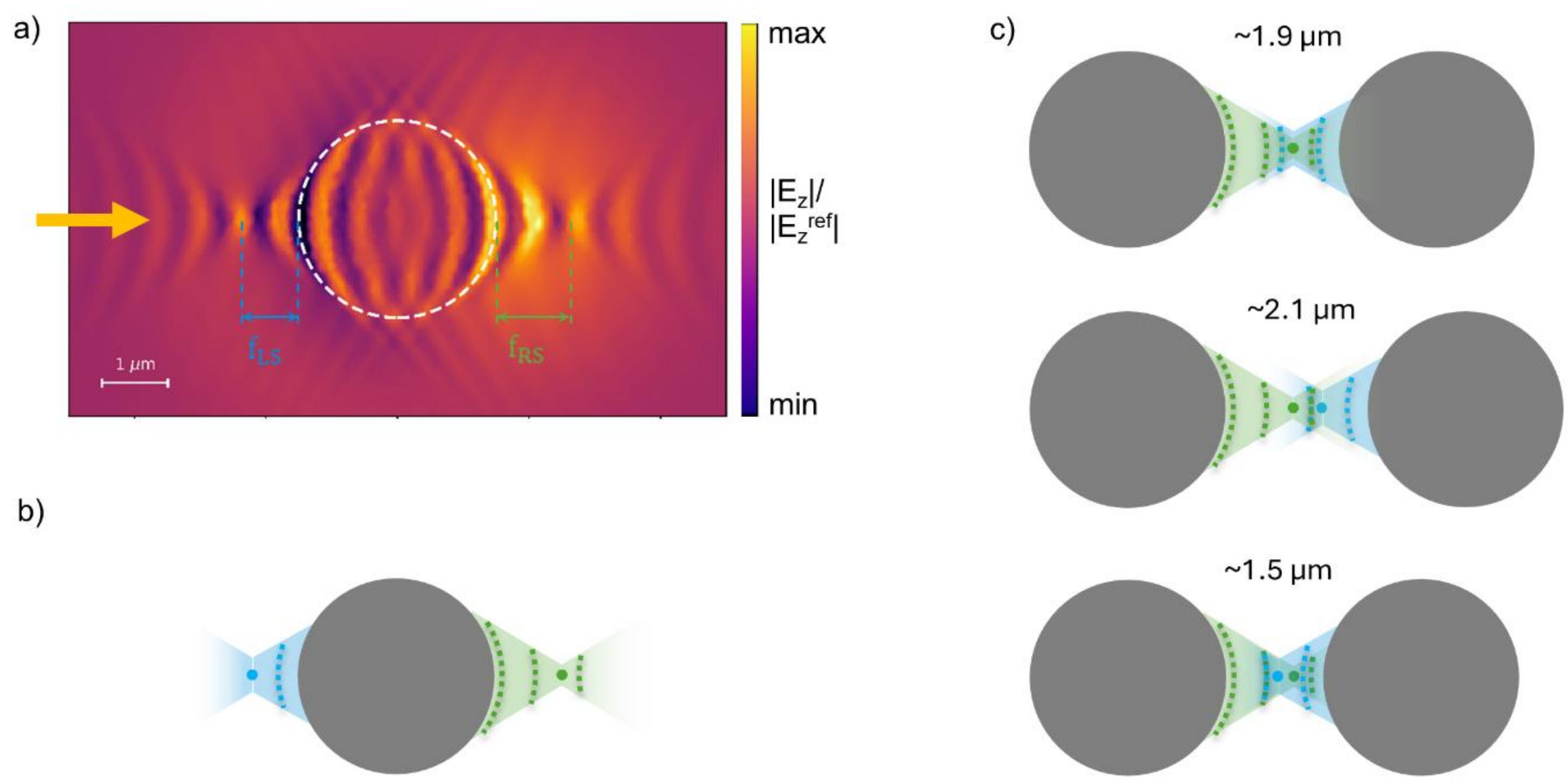

Figure S7: Interference of polaritons launched by two opposing focusing disks. *a) Simulation of a single launching disk at 930 cm^{-1}. The illumination is considered to be from the left side. The scale bar is 1 µm. b) Sketch of polaritons launched and focused to both sides of the crystalline disk. c) Sketch of the interference of polaritons launched by two opposing focusing disks for different disk distances. The wave fronts are indicated by dashed curved lines and the focusing is indicated by the shaded area. The focal points are indicated by a dot. Focusing towards the left is sketched in blue and to the right in green.*

The concept of confining polaritons launched by two opposing focusing disks relies on the interference of the launched polaritonic waves. To understand such effects, we first revisit the simulation of polaritons launched by a single disk as depicted in Figure S7a. Polaritons are launched towards the left and right. On both sides the polaritons are focused on a focal point. However, due to the considered illumination direction from the left (c.f. yellow arrow in Figure S7a), which was chosen to resemble the experiments, the focusing is not symmetric. We observe a shorter focal length on the left side of the disk than on the right side due to the interference with the incident light. Furthermore, the polaritons are launched with opposing phases as on the left side there is a minimum at the disk boundary whereas on the right side there is a maximum at the disk boundary. For simplification, we sketch the different behaviors of

polaritons launched to left (green) and right (blue) in Figure S4b. The envelope of the focusing is indicated by a shaded cone and the wave fronts of the polaritons are indicated by dashed lines. The focal points are indicated by dots.

If we now consider the interference of polaritons launched by two opposing disks, polaritons launched to the right interfere with polaritons launched to the left by the other disk (c.f. sketches in Figure S7c). Although both disks have the same radius, their focal lengths are different due to the illumination direction. The asymmetry observed for the single disk determines the interference of the polaritons and thus the distances of maximal field confinement and enhancement. We find a maximal field confinement at a disk distance of approximately 1.9 µm (see top sketch in Figure S7c). At this disk distance the two focal points overlap. However, due to the opposing launching phases, there is not a constructive interference at the focal point similar to the calculations in ref.[11] for an hyperbolic nanocavity. Hence, the field enhancement is not maximal. Instead, maximal field enhancement is observed at slightly larger or lower distances where constructive interference is achieved, however the focal points do not overlap (c.f. sketches in the bottom of Figure S7c).

In summary, the optimal configuration requires perfect spatial overlap of the two focal spots, together with constructive interference when both spots coincide with a field maximum (bright fringe in simulation images shown in Figure S7a), thus maximizing field enhancement and confinement. At a fixed illumination frequency, the radius of the second disk (in an asymmetric configuration) can be adjusted such that both the right-side focal point of the left disk and the left-side focal point of the right disk coincide with a field maximum. Then the distance between the two disks can be designed to achieve perfect spatial overlap of the two focal spots. Many other parameters play a crucial role. For example, the launching phase depends on the illumination direction (c.f. Figure S7a). Furthermore, polariton properties such as the confinement, governed by the illumination frequency (see Supplementary Note 11) and flake

thickness, as well as the geometry of the focusing structure, which determines contribution of high wavevector components reaching the focal spots, strongly influence the resulting interference pattern. We envision our IST platform as a versatile platform for systematically studying these parameters and disentangling their individual contribution owing to the ease of structure fabrication demonstrated in our work.

Our work is the first experimental approach using a double disk geometry for confining in-plane hyperbolic PhPs in α-MoO_3. Remarkably, we could demonstrate enhanced confinement of a factor of 2 compared to the previously shown single disk geometry. Therefore, the double disk geometry might already serve as a nanocavity for in-plane hyperbolic polaritons. As mentioned in the main text, our geometry was inspired by Álvarez-Pérez et al.[11] who introduced the term *nanoresonator* for in-plane hyperbolic PhP in a geometry of an etched α-MoO_3 flake resembling a hyperbolic shape, with synonymous usage of the term *cavity*. The authors argued that such a structure would lead to back-reflection of the propagating polaritons to a single point, thus yielding a hyperbolic nanoresonator for a point source. Note, the authors neglected far-field illumination which is present in our experiments. In contrast to Álvarez-Pérez et al., we do not have a physical etched flake and two IST disks instead of a hyperbolic boundary.

Supplementary Note 11: Tailoring confinement and field enhancement by varying illumination frequency

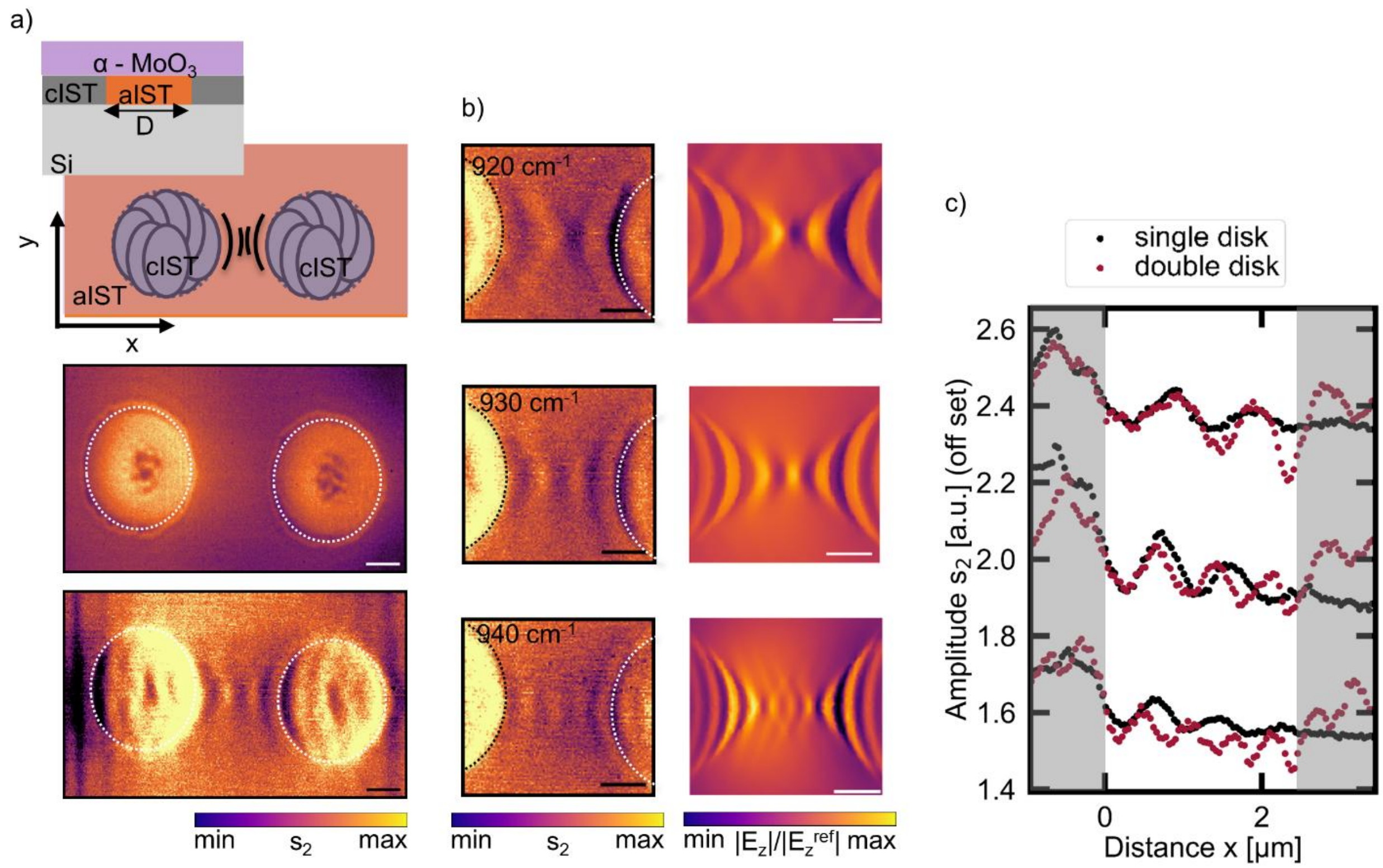

Figure S8: Optically programmed focusing structures with matching focal point to confine phonon polaritons in α-MoO₃. *a) Sketch of the two circular crystalline launching structures of distance 1.9 µm programmed into IST below α-MoO₃ as side (top) and top (middle) view together with an s-SNOM amplitude image at 970 cm^{-1}(top) and at 930 cm^{-1}(bottom). b) s-SNOM amplitude images and simulations of the two focusing disks for three different frequencies increasing from top to bottom. The scale bars correspond to 1 µm. c) Line profiles of a single launching disk (in black) extracted from s-SNOM images in Figure 2d) and line profiles of two launching disks whose focal points overlap (in red) extracted from s-SNOM images in b).*

Tailoring of confinement and enhancement of polaritons between two disks can also be achieved by tuning the incident illumination frequency. We imaged the double disk structure with a distance of 1.9 µm shown in Figure 4 in the main text at three different frequencies and compare it to the respective simulations (c.f. Figure S8b). As the polariton wavelength and the focal length depend on the frequency also the interference pattern is tailored by changing the frequency. For quantification we extract line profiles and compare them to line profiles extracted from single disk at the same frequencies in Figure S8c. With increasing frequency, the polariton

wavelength decreases. Consequently, in the case of the double disks we observe an increasing number of maxima between the disks. At 920 cm^{-1} two maxima can be observed, at 930 cm^{-1} three and at 940 cm^{-1} four maxima. Comparing single and double disk for all frequencies the maxima of the single disks appear broader than that of the double disks indicating an increased confinement in case of the double disks.

Supplementary Note 12: Derivation of the dispersion relation of highly confined PhPs in the investigated layer stack

In this section we show how to obtain the dispersion relation of highly confined polaritons in the used layer stack comprising of the silicon substrate (0), the amorphous IST thin film (1), the $ZnS:SiO_2$ capping layer (2), the α-MoO_3 flake (3), and air as superstrate (4). Since in the high-momentum approximation the contribution of the ordinary modes can be neglected [26], we only consider $\boldsymbol{p}$ modes for the superstrate and substrate and $\boldsymbol{e}$ modes for the biaxial layers. Following the same notation as in ref.[26], the boundary conditions for the tangential components of the electric and magnetic fields can be written as follows:

$$\begin{aligned} a_p^{4\uparrow} e^{iq_{4,p,z}(d_1+d_2+d_3)} \boldsymbol{p}_4 &= \left(a_{e_2}^{3\uparrow} e^{iq_{3,e,z}(d_1+d_2+d_3)} + a_{e_2}^{3\downarrow} e^{-iq_{3,e,z}(d_1+d_2+d_3)}\right) \boldsymbol{e}_3 \\ a_p^{4\uparrow} e^{iq_{4,p,z}(d_1+d_2+d_3)} \Upsilon_p^4 \boldsymbol{p}_{4,+} &= a_{e_2}^{3\uparrow} e^{iq_{3,e,z}(d_1+d_2+d_3)} \boldsymbol{e'}_{3,+} + a_{e_2}^{3\downarrow} e^{-iq_{3,e,z}(d_1+d_2+d_3)} \boldsymbol{e'}_{3,-} \end{aligned} \quad \text{(S2)}$$

$$\begin{aligned} \left(a_{e_2}^{3\uparrow} e^{iq_{3,e,z}(d_1+d_2)} + a_{e_2}^{3\downarrow} e^{-iq_{3,e,z}(d_1+d_2)}\right) \boldsymbol{e}_3 &= \left(a_{e_2}^{2\uparrow} e^{iq_{2,e,z}(d_1+d_2)} + a_{e_2}^{2\downarrow} e^{-iq_{2,e,z}(d_1+d_2)}\right) \boldsymbol{e}_2 \\ a_{e_2}^{3\uparrow} e^{iq_{3,e,z}(d_1+d_2)} \boldsymbol{e'}_{3,+} + a_{e_2}^{3\downarrow} e^{-iq_{3,e,z}(d_1+d_2)} \boldsymbol{e'}_{3,-} &= a_{e_2}^{2\uparrow} e^{iq_{2,e,z}(d_1+d_2)} \boldsymbol{e'}_{2,+} + a_{e_2}^{2\downarrow} e^{-iq_{2,e,z}(d_1+d_2)} \boldsymbol{e'}_{2,-} \end{aligned} \quad \text{(S3)}$$

$$\begin{aligned} \left(a_{e_2}^{2\uparrow} e^{iq_{2,e,z}d_1} + a_{e_2}^{2\downarrow} e^{-iq_{2,e,z}d_1}\right) \boldsymbol{e}_2 &= \left(a_{e_2}^{1\uparrow} e^{iq_{1,e,z}d_1} + a_{e_2}^{1\downarrow} e^{-iq_{1,e,z}d_1}\right) \boldsymbol{e}_1 \\ a_{e_2}^{2\uparrow} e^{iq_{2,e,z}d_1} \boldsymbol{e'}_{2,+} + a_{e_2}^{2\downarrow} e^{-iq_{2,e,z}d_1} \boldsymbol{e'}_{2,-} &= a_{e_2}^{1\uparrow} e^{iq_{1,e,z}d_1} \boldsymbol{e'}_{1,+} + a_{e_2}^{1\downarrow} e^{-iq_{1,e,z}d_1} \boldsymbol{e'}_{1,-} \end{aligned} \quad \text{(S4)}$$

$$\begin{aligned} \left(a_{e_2}^{1\uparrow} + a_{e_2}^{1\downarrow}\right) \boldsymbol{e}_1 &= a_p^{0\downarrow} \boldsymbol{p}_0 \\ a_{e_2}^{1\uparrow} \boldsymbol{e'}_{1,+} + a_{e_2}^{1\downarrow} \boldsymbol{e'}_{1,-} &= a_p^{0\downarrow} \Upsilon_\beta^0 \boldsymbol{p}_0 \end{aligned} \quad \text{(S5)}$$

Eqs. (S2-S5) stand for the boundary conditions at the interfaces between the superstrate (4) and the top (3) layer, between the top (3) and the middle (2) layers, between the middle (2) and the bottom (1) layers, and between the bottom (1) layer and the substrate (0), respectively. Next, we project Eqs. (S2-S5) onto the subvectors $\boldsymbol{p}$. Considering the approximations $\boldsymbol{p} \cdot \boldsymbol{e}_j = 1$ and $\boldsymbol{p} \cdot \boldsymbol{e'}_{j,\pm} = \frac{\varepsilon_{j,z} q_{j,e,z}}{\pm i q_{j,o,z}^2}$, we rewrite Eqs. (S2-S5) as follows:

$$\begin{aligned} a_p^{4\uparrow} e^{iq_{4,p,z}(d_1+d_2+d_3)} &= a_{e_2}^{3\uparrow} e^{iq_{3,e,z}(d_1+d_2+d_3)} + a_{e_2}^{3\downarrow} e^{-iq_{3,e,z}(d_1+d_2+d_3)} \\ -a_p^{4\uparrow} e^{iq_{4,p,z}(d_1+d_2+d_3)} \frac{\varepsilon_4}{iq} &= \frac{\varepsilon_{3,z} q_{3,e,z}}{iq^2} \left(a_{e_2}^{3\uparrow} e^{iq_{3,e,z}(d_1+d_2+d_3)} - a_{e_2}^{3\downarrow} e^{-iq_{3,e,z}(d_1+d_2+d_3)}\right) \end{aligned} \quad \text{(S6)}$$

$$
\begin{gathered}
a_{e_2}^{3\uparrow} e^{iq_{3,e,z}(d_1+d_2)} + a_{e_2}^{3\downarrow} e^{-iq_{3,e,z}(d_1+d_2)} = a_{e_2}^{2\uparrow} e^{iq_{2,e,z}(d_1+d_2)} + a_{e_2}^{2\downarrow} e^{-iq_{2,e,z}(d_1+d_2)} \\
\frac{\varepsilon_{3,z} q_{3,e,z}}{iq^2}\left(a_{e_2}^{3\uparrow} e^{iq_{3,e,z}(d_1+d_2)} - a_{e_2}^{3\downarrow} e^{-iq_{3,e,z}(d_1+d_2)}\right) = \frac{\varepsilon_{2,z} q_{2,e,z}}{iq^2}\left(a_{e_2}^{2\uparrow} e^{iq_{2,e,z}(d_1+d_2)} - a_{e_2}^{2\downarrow} e^{-iq_{2,e,z}(d_1+d_2)}\right)
\end{gathered} \quad \text{(S7)}
$$

$$
\begin{gathered}
a_{e_2}^{2\uparrow} e^{iq_{2,e,z}d_1} + a_{e_2}^{2\downarrow} e^{-iq_{2,e,z}d_1} = a_{e_2}^{1\uparrow} e^{iq_{1,e,z}d_1} + a_{e_2}^{1\downarrow} e^{-iq_{1,e,z}d_1} \\
\frac{\varepsilon_{2,z} q_{2,e,z}}{iq^2}\left(a_{e_2}^{2\uparrow} e^{iq_{2,e,z}d_1} - a_{e_2}^{2\downarrow} e^{-iq_{2,e,z}d_1}\right) = \frac{\varepsilon_{1,z} q_{1,e,z}}{iq^2}\left(a_{e_2}^{1\uparrow} e^{iq_{1,e,z}d_1} - a_{e_2}^{1\downarrow} e^{-iq_{1,e,z}d_1}\right)
\end{gathered} \quad \text{(S8)}
$$

$$
\begin{gathered}
a_{e_2}^{1\uparrow} + a_{e_2}^{1\downarrow} = a_p^{0\downarrow} \\
\frac{\varepsilon_{1,z} q_{1,e,z}}{iq^2}\left(a_{e_2}^{1\uparrow} + a_{e_2}^{1\downarrow}\right) = a_p^{0\downarrow} \frac{\varepsilon_0}{iq}
\end{gathered} \quad \text{(S9)}
$$

For convenience, we multiply the second boundary condition in Eqs. (S6-S9) by iq^2, leading to the following system of equations:

$$
\begin{gathered}
a_p^{4\uparrow} e^{iq_{4,p,z}(d_1+d_2+d_3)} = a_{e_2}^{3\uparrow} e^{iq_{3,e,z}(d_1+d_2+d_3)} + a_{e_2}^{3\downarrow} e^{-iq_{3,e,z}(d_1+d_2+d_3)} \\
-a_p^{4\uparrow} e^{iq_{4,p,z}(d_1+d_2+d_3)} \varepsilon_4 q = \varepsilon_{3,z} q_{3,e,z}\left(a_{e_2}^{3\uparrow} e^{iq_{3,e,z}(d_1+d_2+d_3)} - a_{e_2}^{3\downarrow} e^{-iq_{3,e,z}(d_1+d_2+d_3)}\right)
\end{gathered} \quad \text{(S10)}
$$

$$
\begin{gathered}
a_{e_2}^{3\uparrow} e^{iq_{3,e,z}(d_1+d_2)} + a_{e_2}^{3\downarrow} e^{-iq_{3,e,z}(d_1+d_2)} = a_{e_2}^{2\uparrow} e^{iq_{2,e,z}(d_1+d_2)} + a_{e_2}^{2\downarrow} e^{-iq_{2,e,z}(d_1+d_2)} \\
\varepsilon_{3,z} q_{3,e,z}\left(a_{e_2}^{3\uparrow} e^{iq_{3,e,z}(d_1+d_2)} - a_{e_2}^{3\downarrow} e^{-iq_{3,e,z}(d_1+d_2)}\right) = \varepsilon_{2,z} q_{2,e,z}\left(a_{e_2}^{2\uparrow} e^{iq_{2,e,z}(d_1+d_2)} - a_{e_2}^{2\downarrow} e^{-iq_{2,e,z}(d_1+d_2)}\right)
\end{gathered} \quad \text{(S11)}
$$

$$
\begin{gathered}
a_{e_2}^{2\uparrow} e^{iq_{2,e,z}d_1} + a_{e_2}^{2\downarrow} e^{-iq_{2,e,z}d_1} = a_{e_2}^{1\uparrow} e^{iq_{1,e,z}d_1} + a_{e_2}^{1\downarrow} e^{-iq_{1,e,z}d_1} \\
\varepsilon_{2,z} q_{2,e,z}\left(a_{e_2}^{2\uparrow} e^{iq_{2,e,z}d_1} - a_{e_2}^{2\downarrow} e^{-iq_{2,e,z}d_1}\right) = \varepsilon_{1,z} q_{1,e,z}\left(a_{e_2}^{1\uparrow} e^{iq_{1,e,z}d_1} - a_{e_2}^{1\downarrow} e^{-iq_{1,e,z}d_1}\right)
\end{gathered} \quad \text{(S12)}
$$

$$
\begin{gathered}
a_{e_2}^{1\uparrow} + a_{e_2}^{1\downarrow} = a_p^{0\downarrow} \\
\varepsilon_{1,z} q_{1,e,z}\left(a_{e_2}^{1\uparrow} + a_{e_2}^{1\downarrow}\right) = a_p^{0\downarrow} \varepsilon_0 q
\end{gathered} \quad \text{(S13)}
$$

Next, we start from Eq. (S13) to iteratively solve the system. By dividing the top and bottom boundary conditions in Eq. (S13), we get to

$$
a_{\gamma_1}^{1\downarrow} = \left(\frac{\varepsilon_{1,z} q_{1,e,z} - \varepsilon_s q}{\varepsilon_{1,z} q_{1,e,z} + \varepsilon_s q}\right) a_{\gamma_1}^{1\uparrow} \quad \text{(S14)}
$$

Inserting Eq. (S14) into Eq. (S12), and dividing the two boundary conditions, we get to

$$
\frac{a_{e_2}^{2\uparrow} e^{iq_{2,e,z}d_1} + a_{e_2}^{2\downarrow} e^{-iq_{2,e,z}d_1}}{\varepsilon_{2,z} q_{2,e,z}\left(a_{e_2}^{2\uparrow} e^{iq_{2,e,z}d_1} - a_{e_2}^{2\downarrow} e^{-iq_{2,e,z}d_1}\right)} = D_1 \quad \text{(S15)}
$$

where

$$D_1 = \frac{\varepsilon_{1,z}q_{1,e,z}cos(\xi_{1,e,z}) + i\varepsilon_0 q sin(\xi_{1,e,z})}{\varepsilon_{1,z}q_{1,e,z}\varepsilon_0 q cos(\xi_{1,e,z}) + i(\varepsilon_{1,z}q_{1,e,z})^2 sin(\xi_{1,e,z})} \text{ (S16)}$$

Eq. (S16) leads to

$$a_{e_2}^{2\downarrow} = e^{2iq_{2,e,z}d_1}\left(\frac{D_1\varepsilon_{2,z}q_{2,e,z} - 1}{D_1\varepsilon_{2,z}q_{2,e,z} + 1}\right)a_{e_2}^{2\uparrow} \text{ (S17)}$$

Inserting Eq. (S17) into Eq. (S11), and dividing the two boundary conditions, we get to

$$\frac{a_{e_2}^{3\uparrow}e^{iq_{3,e,z}(d_1+d_2)} + a_{e_2}^{3\downarrow}e^{-iq_{3,e,z}(d_1+d_2)}}{\varepsilon_{2,z}q_{2,e,z}\left(a_{e_2}^{2\uparrow}e^{iq_{2,e,z}(d_1+d_2)} - a_{e_2}^{2\downarrow}e^{-iq_{2,e,z}(d_1+d_2)}\right)} = D_2 \quad \text{(S18)}$$

where

$$D_2 = \frac{D_1\varepsilon_{2,z}q_{2,e,z}cos(\xi_{2,e,z}) + isin(\xi_{2,e,z})}{\varepsilon_{2,z}q_{2,e,z}cos(\xi_{2,e,z}) + iD_1(\varepsilon_{2,z}q_{2,e,z})^2 sin(\xi_{2,e,z})} \text{(S19)}$$

Eq. (S19) leads to

$$a_{e_2}^{3\downarrow} = e^{2iq_{3,e,z}(d_1+d_2)}\left(\frac{D_2\varepsilon_{3,z}q_{3,e,z} - 1}{D_2\varepsilon_{3,z}q_{3,e,z} + 1}\right)a_{e_2}^{3\uparrow} \text{ (S20)}$$

Inserting Eq. (S20) into Eq. (S10), and dividing the two boundary conditions, we get to

$$-\frac{1}{\varepsilon_4 q} = \frac{D_2\varepsilon_{3,z}q_{3,e,z}cos(\xi_{3,e,z}) + isin(\xi_{3,e,z})}{\varepsilon_{3,z}q_{3,e,z}cos(\xi_{3,e,z}) + iD_3(\varepsilon_{3,z}q_{3,e,z})^2 sin(\xi_{3,e,z})} \text{ (S21)}$$

Eq. (S21) can be rewritten as follows:

$$tan(\xi_{3,e,z}) = \frac{\frac{i}{D_2\varepsilon_{3,z}q_{3,e,z}} + \frac{i\varepsilon_4 q}{\varepsilon_{3,z}q_{3,e,z}}}{1 + \frac{\varepsilon_4 q}{D_2(\varepsilon_{3,z}q_{3,e,z})^2}} \text{ (S22)}$$

Using the identity $atan\left(\frac{x+y}{1-xy}\right) = atan(x) + atan(y)$ and noting that $\xi_{3,e,z} = \mp\frac{iqk_0d_3}{\rho_3}$ leads to the following equation describing the dispersion of highly confined polaritons in the investigated layer stack:

$$q = \frac{\rho_3}{k_0 d_3}\left[atan\left(\frac{i\varepsilon_4 q}{\varepsilon_{3,z} q_{3,e,z}}\right) + atan\left(\frac{i}{D_2 \varepsilon_{3,z} q_{3,e,z}}\right) + \pi l\right] \text{ (S23)}$$

Noting that $q_{j,e,z} = \pm i\frac{q}{\rho_j}$, we can rewrite Eq. (S23) as follows:

$$q = \frac{\rho_3}{k_0 d_3}\left[atan\left(\frac{\varepsilon_4 \rho_3}{\varepsilon_{3,z}}\right) + atan\left(\frac{\rho_3}{D_2 \varepsilon_{3,z} q}\right) + \pi l\right] \text{ (S24)}$$

with

$$D_2 = \frac{D_1 \varepsilon_{2,z} q_{2,e,z} cos(\xi_{2,e,z}) + isin(\xi_{2,e,z})}{\varepsilon_{2,z} q_{2,e,z} cos(\xi_{2,e,z}) + D_1 (\varepsilon_{2,z} q_{2,e,z})^2 isin(\xi_{2,e,z})}, \text{(S25)}$$

$$D_1 = \frac{D_0 \varepsilon_{1,z} q_{1,e,z} cos(\xi_{1,e,z}) + isin(\xi_{1,e,z})}{\varepsilon_{1,z} q_{1,e,z} cos(\xi_{1,e,z}) + D_0 (\varepsilon_{1,z} q_{1,e,z})^2 isin(\xi_{1,e,z})} \text{ (S26)}$$

and

$$D_0 = \frac{i}{\varepsilon_0 q} \text{ (S27)}$$

To obtain the out-of-plane Fresnel reflection coefficient, we add to Eq. (S10) a downgoing contribution $a_p^{4\downarrow} e^{iq_{4,p,z}(d_1+d_2+d_3)}$:

$$\begin{gathered} a_p^{4\uparrow} e^{iq_{4,p,z}(d_1+d_2+d_3)} + a_p^{4\downarrow} e^{iq_{4,p,z}(d_1+d_2+d_3)} = a_{e_2}^{3\uparrow} e^{iq_{3,e,z}(d_1+d_2+d_3)} + a_{e_2}^{3\downarrow} e^{-iq_{3,e,z}(d_1+d_2+d_3)} \\ -\varepsilon_4 q\left(a_p^{4\uparrow} e^{iq_{4,p,z}(d_1+d_2+d_3)} - a_p^{4\downarrow} e^{iq_{4,p,z}(d_1+d_2+d_3)}\right) = \varepsilon_{3,z} q_{3,e,z}\left(a_{e_2}^{3\uparrow} e^{iq_{3,e,z}(d_1+d_2+d_3)} - a_{e_2}^{3\downarrow} e^{-iq_{3,e,z}(d_1+d_2+d_3)}\right) \end{gathered} \text{ (S28)}$$

Dividing the boundary conditions in Eq. (S28), we get

$$\frac{a_p^{4\uparrow} e^{iq_{4,p,z}(d_1+d_2+d_3)} + a_p^{4\downarrow} e^{-iq_{4,p,z}(d_1+d_2+d_3)}}{-\varepsilon_3 q\left(a_p^{4\uparrow} e^{iq_{4,p,z}(d_1+d_2+d_3)} - a_p^{4\downarrow} e^{iq_{4,p,z}(d_1+d_2+d_3)}\right)} = D_3 \quad \text{(S29)}$$

where

$$D_3 = \frac{D_2 \varepsilon_{3,z} q_{3,e,z} cos(\xi_{3,e,z}) + isin(\xi_{3,e,z})}{\varepsilon_{3,z} q_{3,e,z} cos(\xi_{3,e,z}) + D_2 (\varepsilon_{3,z} q_{3,e,z})^2 isin(\xi_{3,e,z})} \text{ (S60)}$$

The Fresnel reflection coefficient is found as the quotient

$$r_p = \frac{E_{4,r,z}}{E_{4,i,z}} \quad \text{(S61)}$$

where $E_{4,r,z} = a_p^{4\uparrow} e^{iq_{4,p,z}(d_1+d_2+d_3)}$ and $E_{4,i,z} = a_p^{4\downarrow} e^{-iq_{4,p,z}(d_1+d_2+d_3)}$. It reads, explicitly:

$$r_p = \frac{(D_3 \varepsilon_4 q - 1)}{(D_3 \varepsilon_4 q + 1)} \quad \text{(S62)}$$

This model has been derived following ref.[29].

References

[1] M. Stepanova, S. Dew, *Nanofabrication*, Springer Vienna, Vienna **2012**.

[2] L. Jäckering, A. Moos, L. Conrads, Y. Li, A. Rothstein, D. Malik, K. Watanabe, T. Taniguchi, M. Wuttig, C. Stampfer, T. Taubner, *Nano Letters* **2025**, *25*, 15809.

[3] D. Lu, Y. Zeng, Q. Yan, Q. Chen, W. Ma, X. Luo, M. Xu, X. Yang, P. Li, *Nanophotonics* **2024**, *13*, 937.

[4] N. A. Aghamiri, G. Hu, A. Fali, Z. Zhang, J. Li, S. Balendhran, S. Walia, S. Sriram, J. H. Edgar, S. Ramanathan, A. Alù, Y. Abate, *Nature Communications* **2022**, *13*, 4511.

[5] Y. Qu, N. Chen, H. Teng, H. Hu, J. Sun, R. Yu, D. Hu, M. Xue, C. Li, B. Wu, J. Chen, Z. Sun, M. Liu, Y. Liu, F. J. García de Abajo, Q. Dai, *Advanced Materials* **2022**, *34*, e2105590.

[6] H. Hu, N. Chen, H. Teng, R. Yu, Y. Qu, J. Sun, M. Xue, D. Hu, B. Wu, C. Li, J. Chen, M. Liu, Z. Sun, Y. Liu, P. Li, S. Fan, F. J. García de Abajo, Q. Dai, *Nature Nanotechnology* **2022**, *17*, 940.

[7] J. Liang, J. Zhu, Y. Zhao, S. Wang, Y. Gong, Y. Zhang, G. P. Wang, *ACS Photonics* **2024**, *11*, 5031.

[8] J. Martín-Sánchez, J. Duan, J. Taboada-Gutiérrez, G. Álvarez-Pérez, K. V. Voronin, I. Prieto, W. Ma, Q. Bao, V. S. Volkov, R. Hillenbrand, A. Y. Nikitin, P. Alonso-González, *Science Advances* **2021**, *7*, eabj0127.

[9] Z. Zheng, J. Jiang, N. Xu, X. Wang, W. Huang, Y. Ke, S. Zhang, H. Chen, S. Deng, *Advanced Materials* **2022**, *34*, e2104164.

[10] Y. Zhou, Z. Guo, A. Tarazaga Martín-Luengo, C. Lanza, G. Álvarez-Pérez, C. Yu, C. Li, W. Xia, J. Álvarez Cuervo, X. Duan, Y. Wang, J. Martín-Sánchez, A. Y. Nikitin, Y. Yao, J. Li, P. Alonso-Gonzalez, J. Duan, *Nature Nanotechnology* **2026**, *21*, 23.

[11] G. Álvarez-Pérez, J. Duan, J. Taboada-Gutiérrez, Q. Ou, E. Nikulina, S. Liu, J. H. Edgar, Q. Bao, V. Giannini, R. Hillenbrand, J. Martín-Sánchez, A. Y. Nikitin, P. Alonso-González, *Science Advances* **2022**, *8*, eabp8486.

[12] A. Poddubny, I. Iorsh, P. Belov, Y. Kivshar, *Nature Photonics* **2013**, *7*, 948.

[13] W. Ma, P. Alonso-González, S. Li, A. Y. Nikitin, J. Yuan, J. Martín-Sánchez, J. Taboada-Gutiérrez, I. Amenabar, P. Li, S. Vélez, C. Tollan, Z. Dai, Y. Zhang, S. Sriram, K. Kalantar-Zadeh, S.-T. Lee, R. Hillenbrand, Q. Bao, *Nature* **2018**, *562*, 557.

[14] J. Chen, M. Badioli, P. Alonso-González, S. Thongrattanasiri, F. Huth, J. Osmond, M. Spasenović, A. Centeno, A. Pesquera, P. Godignon, A. Z. Elorza, N. Camara, F. J. García de Abajo, R. Hillenbrand, F. H. L. Koppens, *Nature* **2012**, *487*, 77.

[15] Z. Fei, A. S. Rodin, G. O. Andreev, W. Bao, A. S. McLeod, M. Wagner, L. M. Zhang, Z. Zhao, M. Thiemens, G. Dominguez, M. M. Fogler, A. H. Castro Neto, C. N. Lau, F. Keilmann, D. N. Basov, *Nature* **2012**, *487*, 82.

[16] S. Dai, Q. Ma, Y. Yang, J. Rosenfeld, M. D. Goldflam, A. McLeod, Z. Sun, T. I. Andersen, Z. Fei, M. Liu, Y. Shao, K. Watanabe, T. Taniguchi, M. Thiemens, F. Keilmann, P. Jarillo-Herrero, M. M. Fogler, D. N. Basov, *Nano Letters* **2017**, *17*, 5285.

[17] Achim Woessner, Mark B. Lundeberg, Yuanda Gao, Alessandro Principi, P. Alonso-González, Matteo Carrega, Kenji Watanabe, Takashi Taniguchi, Giovanni Vignale, Marco Polini, James Hone, Rainer Hillenbrand, Frank H. L. Koppens, *Nature Materials* **2015**, *14*, 421.

[18] A. Huber, N. Ocelic, D. Kazantsev, R. Hillenbrand, *Applied Physics Letters* **2005**, *87*, 81103.

[19] S. Dai, Q. Ma, T. Andersen, A. S. McLeod, Z. Fei, M. K. Liu, M. Wagner, K. Watanabe, T. Taniguchi, M. Thiemens, F. Keilmann, P. Jarillo-Herrero, M. M. Fogler, D. N. Basov, *Nature Communications* **2015**, *6*, 6963.

[20] P. Li, M. Lewin, A. V. Kretinin, J. D. Caldwell, K. S. Novoselov, T. Taniguchi, K. Watanabe, F. Gaussmann, T. Taubner, *Nature Communications* **2015**, *6*, 7507.

[21] M. He, G. R. S. Iyer, S. Aarav, S. S. Sunku, A. J. Giles, T. G. Folland, N. Sharac, X. Sun, J. Matson, S. Liu, J. H. Edgar, J. W. Fleischer, D. N. Basov, J. D. Caldwell, *Nano Letters* **2021**, *21*, 7921.

[22] K. Liu, J. Zheng, Y. Sha, B. Lyu, F. Li, Y. Park, Y. Ren, K. Watanabe, T. Taniguchi, J. Jia, W. Luo, Z. Shi, J. Jung, G. Chen, *Nature Nanotechnology* **2024**, *19*, 188.

[23] L. Jäckering, K. G. Wirth, L. Conrads, J. B. Profe, A. Rothstein, H. Kyoseva, K. Watanabe, T. Taniguchi, D. M. Kennes, C. Stampfer, L. Waldecker, T. Taubner, *Advanced Science* **2025**, *12*, e2409039.

[24] J. Duan, A. T. Martín-Luengo, C. Lanza, S. Partel, K. Voronin, A. I. F. Tresguerres-Mata, G. Álvarez-Pérez, A. Y. Nikitin, J. Martín-Sánchez, P. Alonso-González, *Science Advances* **2025**, *11*, eads0569.

[25] Y. Ma, G. Zhong, Z. Dai, Q. Ou, *npj Nanophotonics* **2024**, *1*.

[26] G. Álvarez-Pérez, K. V. Voronin, V. S. Volkov, P. Alonso-González, A. Y. Nikitin, *Physical Review B* **2019**, *100*, 235408.

[27] Z. Zheng, J. Chen, Y. Wang, X. Wang, X. Chen, P. Liu, J. Xu, W. Xie, H. Chen, S. Deng, N. Xu, *Advanced Materials* **2018**, *30*, e1705318.

[28] Z. Zheng, N. Xu, S. L. Oscurato, M. Tamagnone, F. Sun, Y. Jiang, Y. Ke, J. Chen, W. Huang, W. L. Wilson, A. Ambrosio, S. Deng, H. Chen, *Science Advances* **2019**, *5*, eaav8690.

[29] C. Lanza, J. Álvarez-Cuervo, K. V. Voronin, G. Álvarez-Pérez, A. T. Martín-Luengo, J. Martín-Sánchez, A. Y. Nikitin, P. Alonso-González, *Physical Review B* **2026**, *114*, 45423.
